# Vandetanib drives growth arrest and promotes sensitivity to imatinib in chronic myeloid leukemia by targeting ephrin type‐B receptor 4

**DOI:** 10.1002/1878-0261.13270

**Published:** 2022-06-27

**Authors:** Weina Ma, Man Zhu, Bo Wang, Zhengyan Gong, Xia Du, Tianfeng Yang, Xianpeng Shi, Bingling Dai, Yingzhuan Zhan, Dongdong Zhang, Yanhong Ji, Yang Wang, Song Li, Yanmin Zhang

**Affiliations:** ^1^ School of Pharmacy, Health Science Center Xi'an Jiaotong University China; ^2^ State Key Laboratory of Shaanxi for Natural Medicines Research and Engineering Xi'an China; ^3^ Institute of Traditional Chinese Medicine Shaanxi Academy of Traditional Chinese Medicine Xi'an China; ^4^ School of Basic Medical Sciences Xi'an Jiaotong University China; ^5^ Department of Pharmaceutical Sciences, School of Pharmacy, Center for Pharmacogenetics University of Pittsburgh PA USA

**Keywords:** chronic myeloid leukemia, combined treatment, EPHB4, vandetanib

## Abstract

The oncogenic role of ephrin type‐B receptor 4 (EPHB4) has been reported in many types of tumors, including chronic myeloid leukemia (CML). Here, we found that CML patients have a higher *EPHB4* expression level than healthy subjects. *EPHB4* knockdown inhibited growth of K562 cells (a human immortalized myelogenous leukemia cell line). In addition, transient transfection of *EPHB4* siRNA led to sensitization to imatinib. These growth defects could be fully rescued by *EPHB4* transfection. To identify an EPHB4‐specific inhibitor with the potential of rapid translation into the clinic, a pool of clinical compounds was screened and vandetanib was found to be most sensitive to K562 cells, which express a high level of EPHB4. Vandetanib mainly acts on the intracellular tyrosine kinase domain and interacts stably with a hydrophobic pocket. Furthermore, vandetanib downregulated EPHB4 protein via the ubiquitin‐proteasome pathway and inhibited PI3K/AKT and MAPK/ERK signaling pathways in K562 cells. Vandetanib alone significantly inhibited tumor growth in a K562 xenograft model. Furthermore, the combination of vandetanib and imatinib exhibited enhanced and synergistic growth inhibition against imatinib‐resistant K562 cells *in vitro* and *in vivo*. These findings suggest that vandetanib drives growth arrest and overcomes the resistance to imatinib in CML via targeting EPHB4.

## Graphical abstract

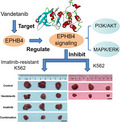

**Abbreviations**
AC: affinity chromatography
BaF3: mouse B lymphocyte cells
CML: chronic myeloid leukemia
Ct: threshold cycle
EGFR: epidermal growth factor receptor
EPHB4: ephrin type‐B receptor 4
GEO: Gene Expression Omnibus
GO: gene ontology
MCs: mononuclear cells
MD: molecular dynamics
NPT: constant pressure and constant temperature
NVT: constant volume and constant temperature
PB: peripheral blood
PDB: protein data bank
Ph: Philadelphia
PVDF: polyvinylidene difluoride
RMSD: root mean square deviation
RMSF: root‐mean‐square fluctuation.
SEM: mean ± standard error of means
SPR: surface plasmon resonance
TBST: Tris‐buffered saline containing 0.05% Tween‐20
TIE2: tyrosine kinase with immunoglobulin and EGF domains‐2
TKI: tyrosine kinase inhibitors
VEGFR‐2: vascular endothelial growth factor receptor 2

## 1 Introduction

Chronic myeloid leukemia (CML) is a myeloproliferative disease which accounts for about 15% of newly diagnosed cases of leukemia in adults [1]. It seriously affects human life and health and is a clinical problem that seriously troubles the medical community. Molecular targeted therapy with the characteristics of high efficiency and low toxicity is widely used in antitumor treatment [2]. The development of targeted drugs will provide new strategies for tumor therapy. However, the lack of targets and drugs makes targeted therapy not beneficial to every patient with tumor.

Ephrin type‐B receptor 4 (EPHB4), a transmembrane tyrosine kinase receptor, is overexpressed and plays an important role in the overgrowth of several epithelial tumors, including colon, prostate, breast and ovarian tumors [3, 4]. EPHB4 has an extracellular region containing an ephrin‐binding domain, a cysteine‐rich domain and two fibronectin type III repeats, and an intracellular region containing a tyrosine kinase domain, a sterile alpha motif and a PDZ‐binding motif [4]. The extracellular region and tyrosine kinase domain represent excellent targets for developing high‐affinity antagonistic compounds [5, 6, 7]. EPHB4 activation can promote cancer growth, migration and metastasis by activating downstream signaling pathways including the RAS/MEK/ERK and EPHB4/RHOA pathways [8, 9]. Therefore, EPHB4 is a promising therapeutic target for the development of novel treatment for various types of cancers [10]. However, little is known about its role in hematologic malignancies.

Chronic myeloid leukemia is characterized by the Philadelphia (Ph) chromosome, which is present in more than 95% of the CML patients [11], and caused by the translocation between chromosome 9 and 22, which leads to the generation of a chimeric gene product named BCR‐ABL [12]. Despite significant progress in developing various tyrosine kinase inhibitors (TKIs), such as imatinib, dasatinib, nilotinib, bosutinib and ponatinib, their clinical impact is limited by the development of drug resistance in CML patients [13, 14, 15]. Tyrosine kinase inhibitors do not completely eradicate CML, but tend to leave most patients in need of lifelong therapy [12, 16, 17]. Recent reports show that EPHB4 is highly expressed in K562 cells [10, 18], and high expression of EPHB4 is associated with imatinib and dasatinib resistance [9, 17, 19], suggesting that EPHB4 may represent a potential target for CML therapy.

To identify an EPHB4 inhibitor with a potential of rapid translation into clinic, a pool of clinically approved TKIs and compounds was screened in this study. Vandetanib (Caprelsa, *N*‐(4‐bromo‐2‐fluorophenyl)‐6‐methoxy‐7‐[(1‐methylpiperidin‐4‐yl)methoxy]quinazolin‐4‐amine) is approved by the United States Food and Drug Administration for the treatment of metastatic medullary thyroid tumor, which is considered as an oral multiple TKI including vascular endothelial growth factor receptor 2 (VEGFR‐2), epidermal growth factor receptor (EGFR), tyrosine kinase with immunoglobulin and EGF domains‐2 (TIE2) and SRC tyrosine kinase families [20, 21]. In this study, whether vandetanib could target EPHB4 was investigated. More importantly, the potential of vandetanib in arresting CML tumor growth and synergistic action with imatinib in inhibiting imatinib‐resistant CML tumor growth were also examined both *in vitro* and *in vivo*.

## 2 Materials and methods

### 2.1 Chemical and reagents

Vandetanib, afatinib, lapatinib, sunitinib, sorafenib and erlotinib (purity ≥ 98%) were purchased from Ange Pharmaceutical (Nanjing, China). NVP‐BHG712 (purity ≥ 98%) was purchased from Sigma‐Aldrich (St. Louis, MO, USA). Iscove's Modified Dulbecco's Medium (IMDM), RPMI 1640 medium, Dulbecco's Modified Eagle Medium (DMEM), trypsin, 3‐(4,5‐dimethylthiazol‐2‐yl)‐2.5‐diphenyl‐2H‐tetrazolium bromide (MTT) and dimethylsulfoxide (DMSO) were purchased from Sigma‐Aldrich. The RNAfast200 kit was purchased from Fastagen (Shanghai, China) and lipofectamine 2000 reagent was purchased from Invitrogen (Carlsbad, CA, USA). PrimeScript RT Master Mix Perfect Real Time kit, SYBR® Premix Ex TaqTM II and a Thermal Cycle Dice Real time system were purchased from TaKaRa (DRR036A) Biotechnology (Dalian, China). Annexin V‐FITC apoptosis detection kit was purchased from Beyotime Institute of Biotechnology (Shanghai, China). EPHB4 proteins (ag10042 and ag16996) were from Proteintech (Wuhan, China). EPHB4 kinase was obtained from Carna Biosciences (Kobe, Japan). Kinase‐Glo Plus luminescence kinase assay kit was from Promega (Madison, WI, USA). EPHB4 lentivirus was from VectorBuilder Inc. (Guangzhou, China).

The information about the antibodies used in western blot assay is shown in Table S1. BCA protein assay reagent kit and SuperSignal® West Pico were purchased from Pierce Biotechnology (Waltham, MA, USA). Protease inhibitor cocktail and phosphatase inhibitor cocktail were purchased from Roche (Basel, Switzerland).

### 2.2 Animals

Six to eight weeks male BALB/c‐nu mice (the Experimental Animal Center of Xi'an Jiaotong University, Xi'an, China) were used for all *in vivo* experiments. Mice were housed in a specific pathogen‐free laboratory animal room. The methods were carried out in accordance with the approved guidelines of the department of biomedical ethics committee of Xi'an Jiaotong University (No. 2019–966).

### 2.3 Cell lines and cell culture

K562, JLTRG, H9 and MEG‐01 cells were from Shanghai Institute of Cell Biology in the Chinese Academy of Sciences (Shanghai, China). Murine FDC‐P1 hematopoietic cells were from Institute of Basic Medical Sciences, Chinese Academy of Medical Sciences (Beijing, China). Imatinib‐resistant K562 cell line was from Anhui Provincial Hospital of Anhui Medical University (Anhui, China). BaF3 cells and BaF3‐EPHB4 cells from Precedo (Hefei, Anhui). Cells from no more than three passages were used. All cell lines were regularly tested for *Mycoplasma* contamination.

K562 cells were cultured in IMDM containing 10% fetal bovine serum, 100 U·mL^−1^ penicillin and 100 U·mL^−1^ streptomycin. JLTRG and H9 cells were cultured in RPMI 1640 medium containing 10% fetal bovine serum, 100 U·mL^−1^ penicillin and 100 U·mL^−1^ streptomycin. MEG‐01 cells were cultured in DMEM containing 10% fetal bovine serum, 100 U·mL^−1^ penicillin and 100 U·mL^−1^ streptomycin. FDC‐P1 cells were cultured in DMEM containing 10% fetal bovine serum, 25% mouse IL‐3 culture supplement, 100 U·mL^−1^ penicillin and 100 U·mL^−1^ streptomycin. BaF3 cells were cultured in RPMI 1640 medium supplemented with 10% fetal bovine serum, 100 U·mL^−1^ penicillin, 100 U·mL^−1^ streptomycin and 2 ng·mL^−1^ murine IL‐3 (R&D Systems, Inc., Minneapolis, MN, USA). Cells were grown at 37 °C in a humidified atmosphere with 5% CO_2_.

### 2.4 Apparatus and chromatographic conditions

Ephrin type‐B receptor 4 affinity chromatography [22] was used to investigate whether vandetanib could bind with EPHB4. Chromatographic analysis was performed on a Shimadzu LC‐20A apparatus (Shimadzu, Kyoto, Japan). The data were acquired by using the lc solution software (Shimadzu). Silica gel (ZEX‐II, 5 μm, 100 mesh) was from Qingdao Meigao Chemical Co. Ltd (Qingdao, China). The detection wavelength for the drugs was 247 nm. Mobile phase buffer for chromatography study was 20 mm phosphate buffer (pH 7.4). Flow rate was 0.2 mL·min^−1^ and temperature was 37 °C.

### 2.5 Cell growth assay

Exponential‐phase cells were seeded into 96‐well plates at a density of 2 × 10^4^ cells per well in complete medium. After 24 h, cells were treated with drugs at indicated concentrations and incubated for 48 h. Fresh medium containing 20 μL MTT solution (5 mg·mL^−1^) was then added to each well. Then plates were incubated for another 4 h at 37 °C. After removing the medium, 150 μL DMSO was added to each well. The absorbance was recorded at 490 nm in the microplate reader and the inhibition ratio (I%) was calculated. The cell growth assay was conducted as described previously [23].

### 2.6 Flow cytometric analysis of apoptosis

Exponential‐phase cells were treated with vandetanib for 48 h, and the cells were then collected, washed and resuspended in PBS. The apoptotic cell death rate was examined with Annexin V‐FITC and PI double staining using the Annexin V‐FITC apoptosis detection kit according to the manufacturer's instructions. After staining the cells with Annexin V‐FITC/PI, flow cytometric analysis was performed and data were analyzed using cell quest software (Becton Dickinson, San Jose, CA, USA). The flow cytometric analysis of apoptosis was conducted as described previously [24].

### 2.7 Western blot analysis

Proteins were extracted using RIPA lysis buffer containing protease and phosphatase inhibitor cocktail on ice. The lysates were centrifuged at 12 500 × ***g*** at 4 °C for 10 min. An equivalent amount of protein was resolved on a 10% SDS/PAGE gel and transferred to polyvinylidene difluoride (PVDF) membranes. The membranes were blocked with Tris‐buffered saline containing 0.05% Tween‐20 (TBST) and 5% low‐fat powdered milk for 1 h. The blot was then incubated with the primary antibody overnight at 4 °C. After washing with TBST for 10 min three times, the blot was incubated with a secondary antibody for 1 h at 37 °C. The blot was washed three times with TBST before being exposed to the SuperSignal West Dura Extended Duration substrate. Band intensity was quantified by densitometric analyses using a densitometer. The western blot assay was conducted as described previously [23].

### 2.8 Kinase assay

All of the enzymatic reactions were conducted at 30 °C for 40 min. The 50 μL reaction mixture contains 40 mm Tris, pH 7.4, 10 mm MgCl_2_, 0.1 mg·mL^−1^ BSA, 1 mm DTT, 10 μm ATP, kinase (50 enzyme used/reaction) and the enzyme substrate [0.2 mg·mL^−1^ Poly (Glu, Tyr)]. The compounds were diluted in 10% DMSO and 5 μL of the dilution was added to a 50 μL reaction so that the final concentration of DMSO is 1% in all of reactions. The assay was performed using Kinase‐Glo Plus luminescence kinase assay kit. It measures kinase activity by quantitating the amount of ATP remaining in solution following a kinase reaction. The luminescent signal from the assay is correlated with the amount of ATP present and is inversely correlated with the amount of kinase activity. The kinase assay was conducted as described previously [25].

### 2.9 RNA extraction and RT‐PCR

The RNA extraction and RT‐PCR assay were conducted as described previously [25]. The primer sequences are shown in Table S2. Melt curve analysis was performed at the end of each PCR to confirm the specificity of the PCR product. Threshold cycle (Ct) values of *EPHB4* in each sample were normalized with the *GAPDH* expression. The ratio of *EPHB4* versus the corresponding *GAPDH* of each sample was determined on the basis of the equation

(1) $$\text{EPHB4}/\text{GAPDH}=2^{\mathrm{Ct}\left(\text{GAPDH}\right)-\mathrm{Ct}\left(\text{EPHB4}\right)}.$$

### 2.10 Plasmid transfection

Ephrin type‐B receptor 4 plasmid extraction was performed according to the instructions of Endo‐free Plasmid DNA Mini Kit I (OMEGA). K562 cells were seeded into 6‐well plates at the density of 2 × 10^5^ per well. After 24 h, transfection of EPHB4 plasmid into K562 cells was performed using Lipofectamine 2000 reagent, according to the manufacturer's instructions. The ratio of Lipofectamine 2000 (μL) to EPHB4 plasmid (μg) was 2 : 1. The EPHB4 expression was determined by RT‐PCR to detect mRNA expression and western blot to detect protein expression.

### 2.11 Adenovirus infection

A double‐stranded siRNA against *EPHB4* and nonspecific siRNA (control siRNA) were from Shanghai GenePharma Co., Ltd. (Shanghai, China). The target sequence of *EPHB4* oligo selected was 5′‐CCUUUGAGGUCACUGCAUUTT‐3′ (sense) and 5′‐AAUGCAGUGACCUCAAAGGTT‐3′ (antisense). The recombinant adenovirus was obtained from Vector Builder (Conrad, TX, USA). Transfection of K562 cells with adenovirus was performed in 10 × 10 cm cell culture dish with 5 × 10^6^ cells per dish in 12 mL IMDM medium containing 10% FBS; 24 h later, adenovirus was added to the dishes at a MOI of 6 and the medium was changed to complete medium and the transfected K562 cells were harvested after 6 days of incubation. The EPHB4 expression was determined by western blot to detect protein expression in stably infected K562 cells.

### 2.12 Frontal analysis

Frontal analysis was used to determine the binding affinity between vandetanib and EPHB4. In this study, each column was equilibrated with the pH 7.4 mobile phase buffer. The mobile phase was then switched to a solution that contained a known concentration of the tested drug in mobile buffer. Each drug solution was continuously applied to the column until a breakthrough curve with a level plateau was produced. The system was later switched back to the mobile phase buffer to elute the retained analyte from the column. The *K*_D_ value is investigated by using frontal analysis [26].

### 2.13 Surface plasmon resonance (SPR) analysis

For the SPR analysis, EPHB4 protein was prepared to 50 μg·mL^−1^ by using PBS buffer. The stock solution of the tested drug was 0.02 m dissolved in DMSO. Working solution of tested drugs was diluted into 20, 10, 5, 1 μm using PBS buffer. Firstly, an NTA chip was installed in the OpenSPRTM instrument and was pumped by using PBS buffer at maximum flow rate. After reaching the signal baseline, 80% isopropanol was injected from the injection port, and the control valve was transferred into ‘inject’. After running 10 s, the control valve was back to ‘load’ for bubble exhaust. When the signal reaches at baseline, the flow rate of running buffer (PBS) was adjusted to 20 μL·min^−1^. Then imidazole solution (250 μL) was used to initialize the sensor surface for three times, and NiCl_2_ solution (40 mm, 250 μL) was injected to charge the NTA. Afterwards, His‐labeled EPHB4 (50 μg·mL^−1^, 250 μL)‐dissolved running buffer was injected to interact for 5 min. The activity of the ligand and the maximum surface binding force were confirmed by injecting a high concentration of the tested drug. Finally, increase HCL solution (PH = 2, 150 μL·min^−1^) was used to remove the sample in the system. The SPR assay was conducted as described previously [26, 27].

### 2.14 Structure preparation for molecular dynamics simulations

The starting structure for the docking and molecular dynamics simulation of EPHB4 was taken from the Protein Data Bank (PDB ID: 3ZEW) [28], and the structure of vandetanib was obtained from the PubChem (PubChem CID: 3081361). The complex structure of EPHB4 and vandetanib was obtained by molecular docking. Docking calculations were carried out using the autodock 4.2 (Maynard Olson, Seattle, WA, USA).

### 2.15 Molecular dynamics (MD) simulations

Molecular dynamics simulations were used to determine the binding site of vandetanib on EPHB4 and its mode of binding. The MD simulations were performed using the amber package version 16. To prepare the topology and coordinate files, the amber ff03 all‐atom force fields [29] were used for the protein atoms, and the antechamber module of amber Tools was used to assign GAFF [30] parameters for vandetanib. All MD simulations were carried out by applying periodic boundary conditions in TIP3P water model with a margin of at least 12 Å from any edge of the box to any atom of the solute molecules. The counter ions Na^+^ were added to neutralize the solvated systems. A 3000‐step minimization (steps 1–1000 using conjugated gradient followed by 2000 steps steepest decent) was first carried out using SANDER module. After minimization, a 500 ps constant volume and constant temperature (NVT) simulation was performed to raise the temperature of the system to 298 K while constraining backbone atoms with a 5 kcal·mol^−1^·Å2^−1^ force constant with reference to the structures. A second 200 ps constant pressure and constant temperature (NPT) simulation at 298 K was performed while constraining backbone atoms with a 2 kcal·mol^−1^·Å2^−1^ force constant with reference to the structures. The system was then equilibrated for 1 ns at 298 K without any constraints. The system is then ready for the 100 ns production run. All the 100 ns MD simulations were in the isobaric isothermal (NPT, *T* = 298 K and *P* = 1 atm) ensemble. The SHAKE algorithm was used to fix bonds involving hydrogen. The PME method [31] was used and the nonbonded cutoff distance was set at 10 Å. The time step was set to 2 fs.

### 2.16 Microarray analysis

K562 cells were plated into 6‐well plates 1 day prior to treatment with vandetanib (0 or 1.56 μm) for 48 h. Total RNA was isolated using Trizol. Differential gene expression profiling was performed using RNA deep sequencing by Gminix (Shanghai, China). Differential expression between two conditions was detected using the DEGSeq R package (1.20.0). RNAseq statistics were analyzed as follows: hisat2 software (Daehwan Kim Group, Dallas, TX, USA) was used to perform similarity analysis by aligning reads derived from sequencing data to the reference genome, which in turn enabled annotation and quantification of genes or transcripts and normalization of read counts. Differential gene expression across samples was then analyzed using edger software (Bioconductor, Reilingen, Germany). For each gene, significance *P*‐value and false discovery rate (FDR) were obtained based on the model of negative binomial distribution. Fold changes of gene expression were also estimated by the edger package. Finally multiple hypothesis testing correction was performed with ¦log2 fold change¦ > 1 and FDR < 0.05.

### 2.17 DNA constructs

pX459 v2.0 (Fig. S1) was used for sgRNAs and CAS9 gene expression. sgRNAs for EPHB4 knockout and point mutation were online‐designed at http://crispr.mit.edu/. ssODNs for target mutation were designed according to Zhang's protocol [32]. Oligos used in this article are listed in Table S3.

### 2.18 Construction of K562 EPHB4‐knockout cell line by CRISPR Cas9

K562 cells were seeded and cultured in 60 × 15 mm dishes with IMDM medium containing 10% fetal bovine serum (ExCell Biology, Shanghai, China). Once the cell confluence reached 70–80%, the medium was replaced by IMDM antibiotic‐free and serum‐reduced medium for 24 h before transfection. By using the ExFect 2000 transfection reagent (Vazyme, Nanjing, China), the cells were transfected with the construct containing both *EPHB4* sgRNA and Cas9 protein‐coding sequences. The stable EPHB4‐knockout cells were then harvested after 2 weeks of puromycin (12 μg·mL^−1^) treatment.

### 2.19 Generation of EPHB4 site‐directed mutagenesis cell line

Ephrin type‐B receptor 4 site‐directed mutagenesis cell lines were generated via first knockout of wild‐type EPHB4 using CRISPR/Cas9 system [33], followed by stable transfection with a plasmid carrying EPHB4 with respective point mutation. Two individual sgRNAs were transfected into K562 cell line and the EPHB4‐knockout cells were obtained via puromycin selection. K562 EPHB4‐knockout cells were seeded and cultured in 60 × 15 mm dishes to reach 70–80% of cell confluence. The medium was then replaced by an IMDM antibiotic‐free and serum‐reduced medium for 24 h before transfection. The K562 EPHB4‐KO cells were subsequently used to generate five sublines with defined mutations in the 12 aa residues via stable transfection with pcDNA3.1 vector carrying EPHB4 with respective point mutation. Five mutagenesis constructs EPHB4‐M1, EPHB4‐M2, EPHB4‐M3, EPHB4‐M4 and EPHB4‐M5 were transfected into K562 EPHB4‐knockout cells by using ExFect 2000 transfection reagent (Vazyme) individually. M1 represents point mutation I14A and G15A; M2 represents point mutation M83A, N85A and G86A; M3 represents point mutation A87Del; M4 represents point mutation S90A and F91A; and M5 represents point mutation R93A and L94A. Site‐directed mutagenesis cells were then harvested after 2 days of incubation.

### 2.20 *In vivo* therapeutic study

In order to evaluate the expression of *EPHB4 in vivo*, BALB/c‐nu mice (4–6 weeks) were inoculated with K562/H9 cells at a density of 2 × 10^7^ cells per mouse by tail vein injection. Seven days later, mice were randomly grouped (*n* = 5) and treated with sodium carboxymethylcellulose and vandetanib (40 mg·kg^−1^) every day by intragastric administration. After 2 weeks, whole blood was taken out of the eye socket of the mice and put into the 1.5‐mL tubes pretreated with heparin. Mononuclear cells (MCs) were isolated by Ficoll‐Paque (GE Healthcare, Waukesha, WI, USA) density gradient centrifugation. Afterwards, the samples were used for detecting the content of *EPHB4 in vivo* by RT‐PCR.

For the *in vivo* therapeutic study, BALB/c‐nu mice (4–6 weeks) were injected subcutaneously with wild‐type K562 cells at a density of 2 × 10^7^ cells (diluted in a mixture containing 100 μL PBS and 100 μL high‐concentration matrix) per mouse into the right flanks. When the tumor volume reached about 80–100 mm^3^, mice were randomly grouped (*n* = 3) and treated with sodium carboxymethylcellulose or vandetanib (40 mg·kg^−1^) every day by intragastric administration. Tumor volumes were calculated every 2 days until day 14 according to the formula: (L*W ^2^)/2 (*L* and *W* are the long and short tumor diameters). And in all the period of treatment, body weight was monitored.

In order to evaluate the synergetic action of vandetanib on imatinib‐resistant K562 tumor, BALB/c‐nu mice (4–6 weeks) were injected subcutaneously with imatinib‐resistant K562 cells at a density of 2 × 10^7^ cells (diluted in a mixture containing 100 μL PBS and 100 μL high‐concentration matrix) per mouse into the right flanks. When the tumor volume reached about 80–100 mm^3^, mice were randomly grouped (*n* = 3) and treated with sodium carboxymethylcellulose, vandetanib alone (40 mg·kg^−1^), imatinib alone (100 mg·kg^−1^) and combination treatment [vandetanib alone (40 mg·kg^−1^) plus imatinib (100 mg·kg^−1^)] every day by intragastric administration. Tumor volumes were calculated every 2 days until day 14 according to the formula: (L*W ^2^)/2 (*L* and *W* are the long and short tumor diameters). And in all the period of treatment, body weight was monitored.

After completing the *in vivo* experiment, tumor tissues were collected. Some tumor tissues were collected in −80 °C for the following western blotting. The other tumor tissues were fixed in 4% paraformaldehyde and then embedded in paraffin for immunohistochemical analysis.

### 2.21 Immunochemistry assay

For immunochemistry assay, the paraffin‐embedded tumor tissues were sectioned into slices at 5 μm using an HM325 Rotary Microtome. Then the slices were deparaffinized in xylene and hydrated in descending grades of alcohol. Afterwards, the sections were pretreated with 0.01 mol·L^−1^ citrate‐buffered saline (pH 6.0) and quenched with 0.3% (v/v) hydrogen peroxide for endogenous peroxidase activity. Then, the sections were washed twice in distilled water for 5 min each and incubated with diluted normal blocking serum at room temperature for 1 h. After that, the sections were incubated with primary antibody diluted in blocking buffer at 4 °C overnight and washed with PBS for three times before incubating with secondary antibody for 20 min at 95 °C. Then the tissues were washed with PBS and S‐A/HRP working solution. The tissues were incubated for 1 min in diaminobenzidine tetra‐hydrochloride to develop the peroxidase labeling. Finally, counterstaining was conducted with hematoxylin for imaging.

### 2.22 Statistical analysis

All values are expressed as the mean ± standard error of means (SEM). Three independent experiments were conducted for each assay. Data were compared using the Student's *t*‐test or analysis of variance (ANOVA; SPSS statistical package 16.0, Chicago, IL, USA). Significance was determined with Tukey simultaneous *post hoc* test. A *P* < 0.05 is considered to be statistically significant.

## 3 Results

### 3.1 Effect of EPHB4 on CML

**Fig. 1:**
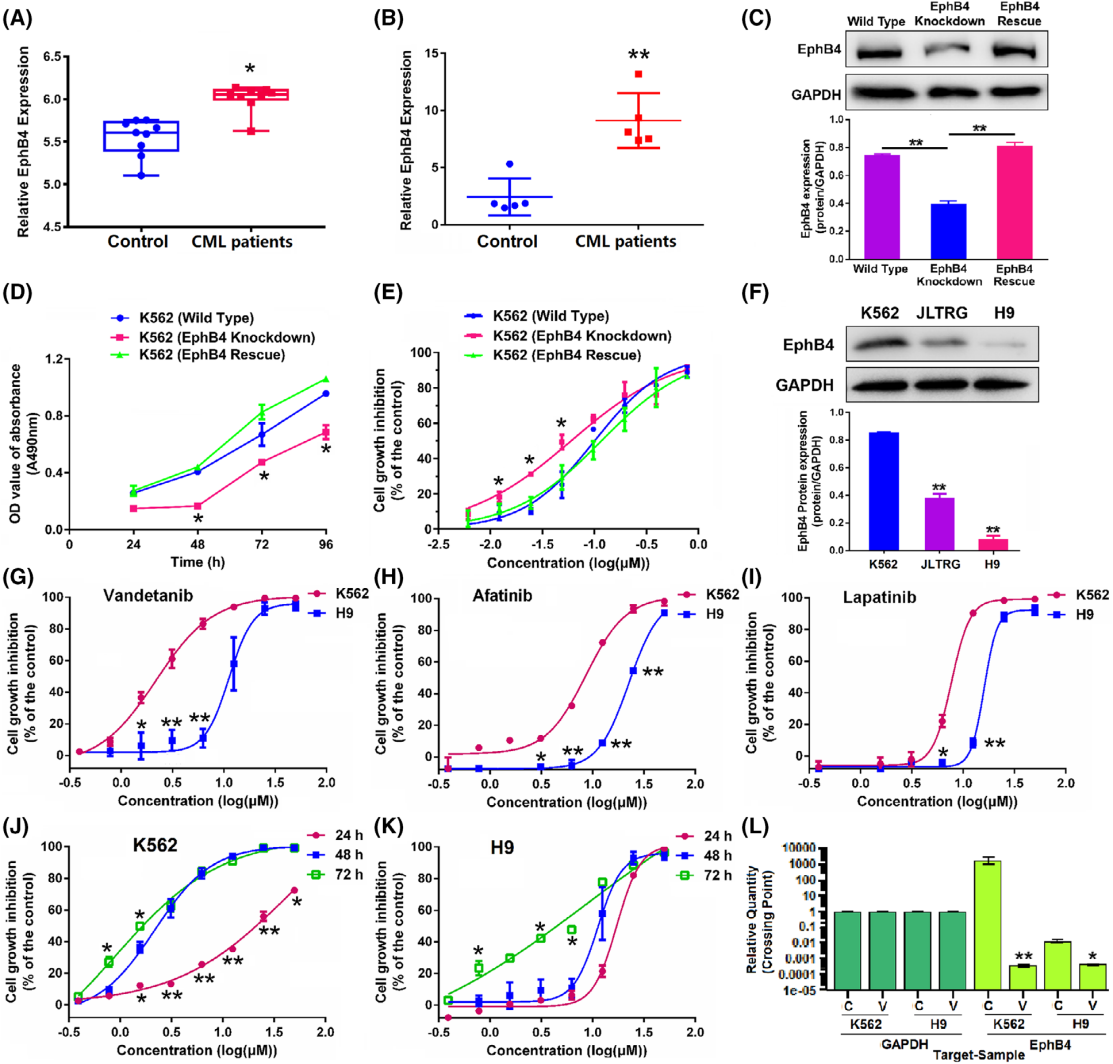
Effect of vandetanib on chronic myeloid leukemia (CML) by targeting ephrin type‐B receptor 4 (EPHB4). (A) The correlation between CML and *EPHB4* from Gene Expression Omnibus (GEO) database (GEO no. GSE33075). *P*‐values were generated by Student's *t*‐test. *P < 0.05 versus the control group. (B) The correlation between CML and *EPHB4* from GEO database (GEO no. GSE100026). *P*‐values were generated by Student's *t*‐test. **P < 0.01 versus the control group. (C) The protein expression of EPHB4 in wild‐type, EPHB4 knockdown and EPHB4 rescue K562 cells. All the results were quantified by densitometry analysis of the bands and normalization to GAPDH. *P*‐values were generated by one‐way ANOVA using the Tukey test for multiple comparisons. **P < 0.01. (D) The growth of wild‐type, EPHB4 knockdown and EPHB4 rescue K562 cells. *P*‐values were generated by one‐way ANOVA using the Tukey test for multiple comparisons. *P < 0.05 versus K562 wild‐type group. (E) Effect of imatinib treatment for 48 h on the growth of wild‐type, EPHB4 knockdown and EPHB4 rescue K562 cells. *P*‐values were generated by one‐way ANOVA using the Tukey test for multiple comparisons. *P < 0.05 versus K562 wild‐type group. (F) Expression levels of EPHB4 protein in K562, JLTRG and H9 cells. All the results were quantified by densitometry analysis of the bands and normalization to GAPDH. *P*‐values were generated by one‐way ANOVA using the Tukey test for multiple comparisons. **P < 0.01 versus the K562 group. (G) Effect of vandetanib treatment for 48 h on cell growth in K562 and H9 cells was determined by MTT assay. *P*‐values were generated by one‐way ANOVA using the Tukey test for multiple comparisons. *P < 0.05, **P < 0.01 versus K562 group. (H) Effect of afatinib treatment for 48 h on cell growth in K562 and H9 cells was determined by MTT assay. *P*‐values were generated by one‐way ANOVA using the Tukey test for multiple comparisons. *P < 0.05, **P < 0.01 versus K562 group. (I) Effect of lapatinib treatment for 48 h on cell growth in K562 and H9 cells was determined by MTT assay. *P*‐values were generated by one‐way ANOVA using the Tukey test for multiple comparisons. *P < 0.05, **P < 0.01 versus K562 group. (J) Effect of vandetanib on cell growth in K562 cells in 24, 48 and 72 h. *P*‐values were generated by one‐way ANOVA using the Tukey test for multiple comparisons. *P < 0.05, **P < 0.01 versus 48 h group. (K) Effect of vandetanib on cell growth in H9 cells in 24, 48 and 72 h. *P*‐values were generated by one‐way ANOVA using the Tukey test for multiple comparisons. *P < 0.05 versus 48 h group. (L) The mRNA expression of *EPHB4* in blood samples of BALB/c‐nu mice inoculated with K562 and H9 cells by tail vein injection treated with sodium carboxymethylcellulose (marked as C) and vandetanib (marked as V, 40 mg·kg^−1^) every day by intragastric administration for 2 weeks. *P*‐values were generated by one‐way ANOVA using the Tukey test for multiple comparisons. *P < 0.05, **P < 0.01 versus sodium carboxymethylcellulose (marked as C) group. The values represent the average of three independent experiments. Data represent the means ± SEM (*n* = 3).

To establish a role of EPHB4 in CML, the expression of *EPHB4* in CML patients was analyzed using the data from GEO database (GEO No. GSE33075 and No. GSE100026). As shown in Fig. 1A,B, the mRNA levels of *EPHB4* were significantly higher in CML patients than in the control subjects. In order to test the effect of EPHB4 on the tumor formation, an EPHB4 stable knockdown K562 cell line and EPHB4 rescue K562 cell line were established (Fig. 1C). Knockdown of EPHB4 expression in K562 cells by shRNA‐mediated gene silencing led to significant slow‐down of cell growth *in vitro* (Fig. 1D). In addition, transient transfection of *EPHB4* siRNA led to sensitization to imatinib (Fig. 1E), and the IC_50_ of imatinib in *EPHB4* siRNA‐ and control siRNA‐transfected cells were 65.35 and 99.14 nm, respectively. Furthermore, these growth defects could be fully rescued by transfection of EPHB4 (Fig. 1D,E).

### 3.2 Vandetanib inhibited the cell growth with various expressed EPHB4 and decreased the *EPHB4* mRNA expression *in vivo*

As shown in Fig. 1F, K562 cells showed a higher protein level of EPHB4 than JLTRG and H9 cells. To identify an EPHB4‐specific inhibitor with a potential of rapid translation into clinic, a pool of clinically approved TKIs and compounds was screened in this study (Fig. S2 and Table S4). The inhibitory effects of vandetanib, afatinib and lapatinib on different cell lines were positively correlated with the expression levels of EPHB4 (Fig. 1G–I). The IC_50_ values of the three drugs in K562 and H9 cells are shown in Table S5. Results showed that vandetanib was most sensitive to K562 cells which express a high level of EPHB4. Moreover, vandetanib exhibited no obvious toxicity in untransformed myeloid cell (FDC‐P1) growth rather than afatinib and lapatinib (Fig. S3). We then further examined the growth inhibition effect of vandetanib in K562 and H9 cells following treatment for different periods of time. As shown in Fig. 1J,K, vandetanib inhibited the growth of both cancer cell lines in a dose‐ and time‐dependent manner. The IC_50_ values of vandetanib in K562 and H9 cells following treatment for 24, 48 and 72 h are summarized in Table S6. Again, vandetanib was more effective in suppressing the growth of K562 cells that express a high level of EPHB4. Moreover, another CML cell line (MEG‐01) was used to evaluate the effect of vandetanib on CML cell growth. Results in Fig. S4 showed that vandetanib effectively inhibited the MEG‐01 cell growth in a dose‐ and time‐dependent manner, and the IC_50_ values are shown in Table S6. To investigate whether vandetanib drives growth arrest by regulating *EPHB4* expression, BALB/c‐nu mice were inoculated with K562 and H9 cells by tail vein injection, and the blood samples were then examined. As shown in Fig. 1L, the mRNA expression of *EPHB4* in blood samples was much higher in K562 model than in H9 model, which was consistent with the *in vitro* results. In addition, treatment with vandetanib led to a downregulation in the mRNA expression level of *EPHB4 in vivo*.

### 3.3 EPHB4 is a therapeutic target for vandetanib

**Fig. 2:**
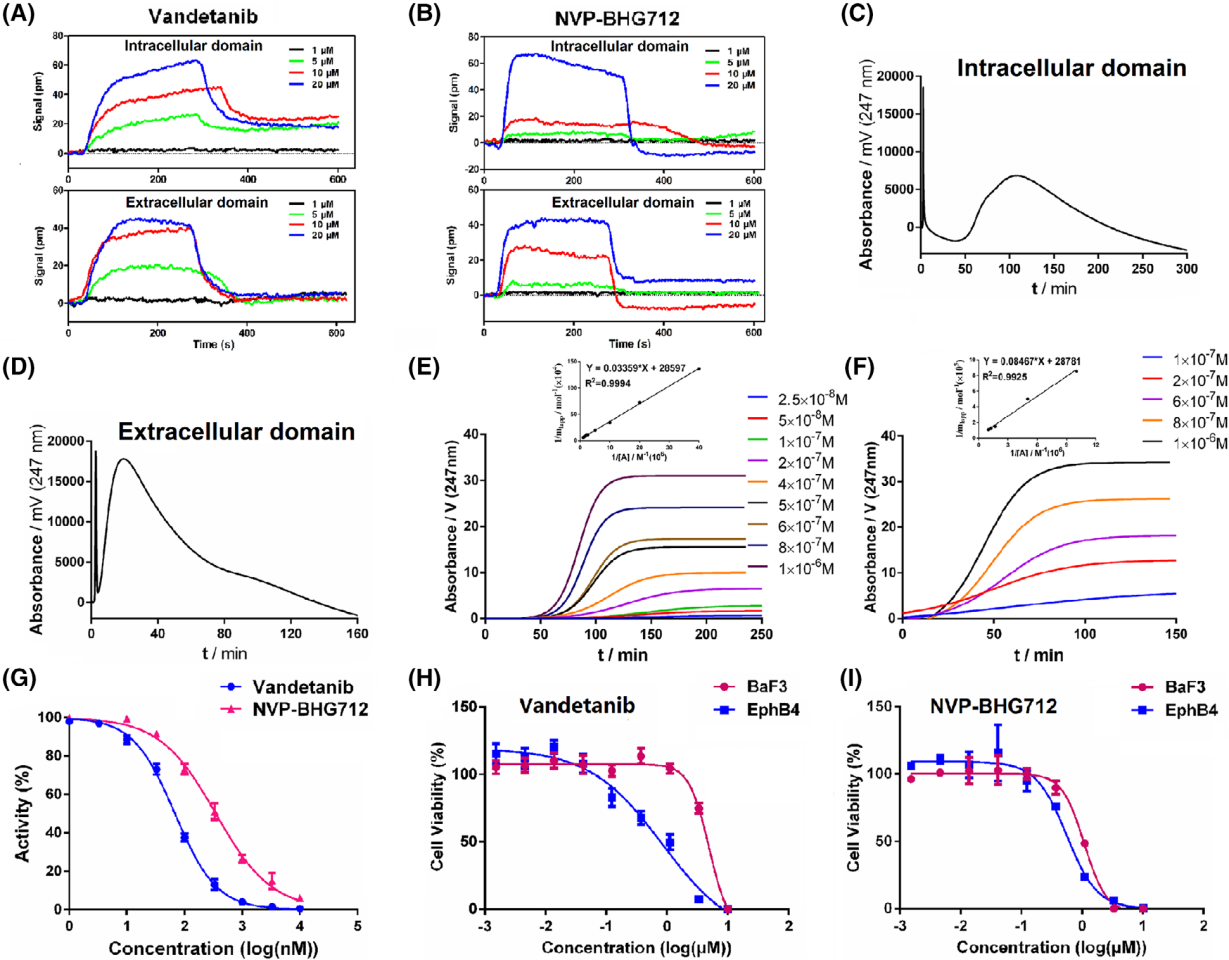
Effect of vandetanib on ephrin type‐B receptor 4 (EPHB4). (A) Binding affinity of vandetanib with the intracellular domain and extracellular domain of EPHB4 by surface plasmon resonance (SPR) analysis. (B) Binding affinity of NVP‐BHG712 with the intracellular domain and extracellular domain of EPHB4 by SPR analysis. (C) The chromatogram of vandetanib on the intracellular domain of EPHB4 affinity column. (D) The chromatogram of vandetanib on the extracellular domain of EPHB4 affinity column. (E) The intracellular domain of EPHB4 affinity chromatographic breakthrough curves of vandetanib and the regression curves achieved by plotting m_Lapp_ versus 1/[A]. (F) The extracellular domain of EPHB4 affinity chromatographic breakthrough curves of vandetanib and the regression curves achieved by plotting m_Lapp_ versus 1/[A]. EPHB4 affinity column 10 × 2.0 mm; flow rate 0.2 mL·min^−1^; column temperature 37 °C; mobile phase 20 mm phosphate‐buffered saline; pH 7.4; detection wavelength 247 nm. (G) Effect of vandetanib and NVP‐BHG712 on EPHB4 kinase activity. (H) Effect of vandetanib treatment for 48 h on the growth of BaF3 cells and BaF3‐EPHB4 cells. (I) Effect of NVP‐BHG712 treatment for 48 h on the growth of BaF3 cells and BaF3‐EPHB4 cells. The values represent the average of three independent experiments. Data represent the means ± SEM (*n* = 3).

We next went on to study the interaction of vandetanib with EPHB4 and identify the EPHB4‐binding domain by affinity chromatography (AC) with an EPHB4 protein affinity column [22] and SPR. An extracellular domain (ag16996, active amino acid sequence 16–539) and an intracellular domain (ag10042, active amino acid sequence 561–987) of EPHB4 were constructed, respectively. NVP‐BHG712, a small molecule EPHB4 kinase‐specific inhibitor, was used as a positive control [34]. The binding affinity of TKIs (vandetanib, afatinib, lapatinib and NVP‐BHG712) with EPHB4 was evaluated by SPR analysis as shown in Fig. 2A,B and Fig. S5. The *K*_D_ values of the TKIs and NVP‐BHG712 binding to the intracellular domain and extracellular domain of EPHB4 are summarized in Table S7. Vandetanib binds with a significantly higher affinity to EPHB4, especially its intracellular domain compared to the other two TKIs and NVP‐BHG712. The retention time of vandetanib on the intracellular domain of EPHB4 affinity column was about 110.22 min, whereas its retention time on the extracellular domain of EPHB4 affinity column was approximately 19.47 min (Fig. 2C,D). Frontal analysis was then used to examine the overall binding of vandetanib to EPHB4. The representative breakthrough curves of vandetanib on the intracellular domain and extracellular domain of EPHB4 affinity columns are shown in Fig. 2E,F. According to Eqn (1), the *K*_D_ values of the binding of vandetanib to the intracellular domain and extracellular domain of EPHB4 were 1.18 and 2.94 μm, respectively. Furthermore, we examined the inhibitory effects on the EPHB4 kinase activity of the three TKIs (vandetanib, afatinib and lapatinib) and NVP‐BHG712 (Fig. 2G and Fig. S6) and their IC_50_ values are shown in Table S8. As shown in Fig. 2G, vandetanib altered EPHB4 kinase activity in a dose‐dependent manner and the effect of vandetanib (IC_50_, 68.33 nm) was much more dramatic than NVP‐BHG712 (IC_50_, 349.30 nm).

To further establish EPHB4 as the therapeutic target in vandetanib treatment, the drug response was also tested in mouse B lymphocyte cells (BaF3) and in EPHB4‐BaF3 cells that stably expressed a high level of EPHB4. EPHB4‐BaF3 subline was established via Gene Engineering Technology (Using wild‐type BaF3 as a motherboard, TEL/BCR/NPM and other genes were fused into the catalytic region of the target gene to induce cells to break away from the dependence on IL‐3 stimulator and to rely on the activity of fusion or mutant activated kinases transferred from outside). As shown in Fig. 2H, vandetanib was much more effective in inhibiting the growth of BaF3‐EPHB4 cells compared to BaF3 cells; the GI_50_ values were 0.51 and 7.71 μm, respectively. Meanwhile, the GI_50_ values of NVP‐BHG712 in BaF3‐EPHB4 and BaF3 cells were 0.56 and 1.48 μm, respectively (Fig. 2I).

### 3.4 EPHB4 signaling pathways involved in the regulation of K562 cells treated by vandetanib

**Fig. 3:**
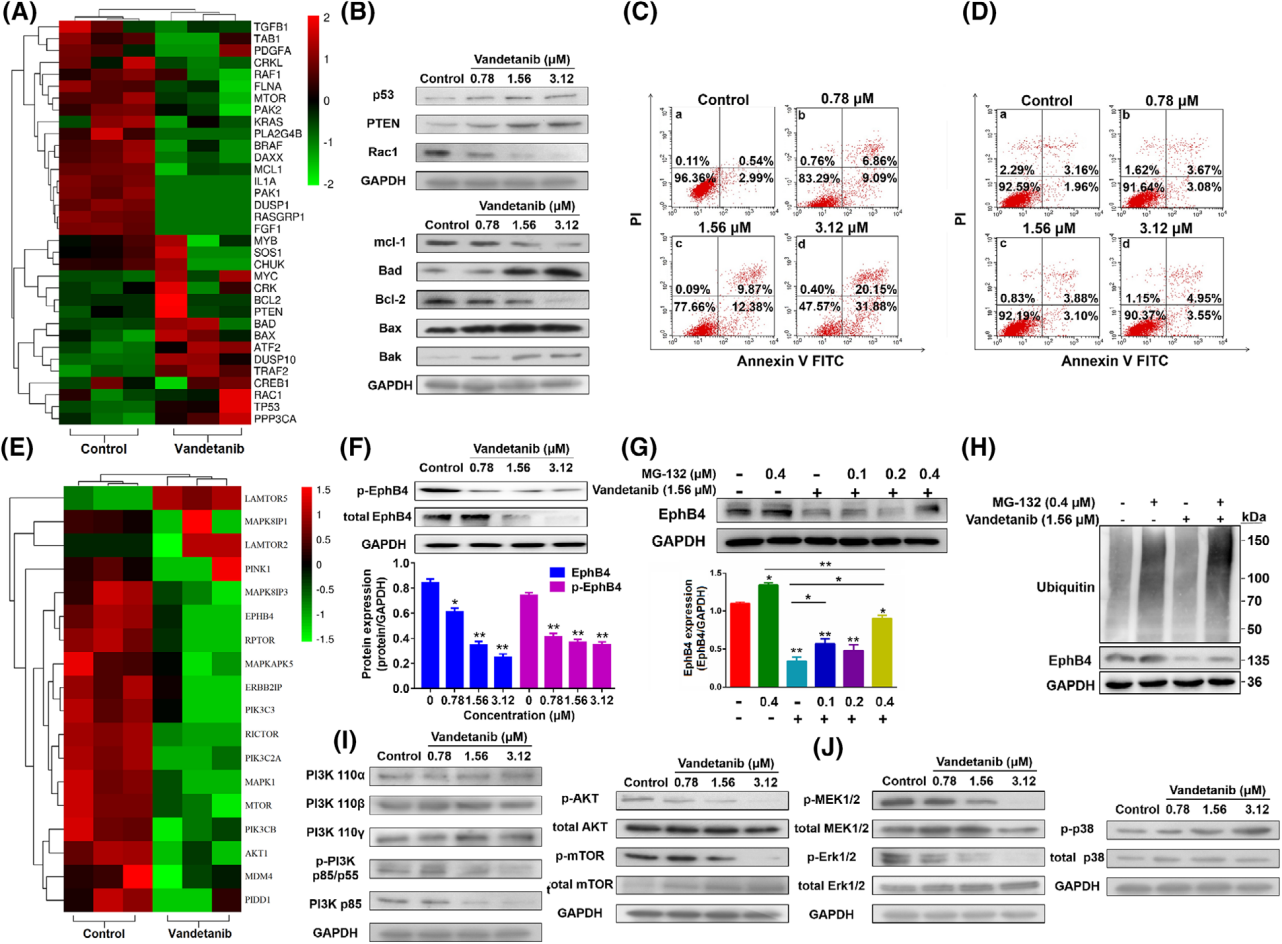
The role of ephrin type‐B receptor 4 (EPHB4) in hematologic tumor cells treated by vandetanib. (A) Hierarchical cluster analysis of the transcriptionally different populations about related molecules of apoptosis in K562 cells with treatment with control or vandetanib for 48 h. Each column represents one sample, and each mRNA was depicted by one row, where red denotes an increase in mRNA expression and green denotes a decrease in mRNA expression as compared with the other group. (B) Effect of vandetanib treatment for 48 h on cell apoptosis‐related protein expression. (C) Effect of vandetanib treatment for 48 h on cell apoptosis in wild‐type K562 cells. (D) Effect of vandetanib treatment for 48 h on cell apoptosis in wild‐type H9 cells. (E) Hierarchical cluster analysis of the transcriptionally different populations about related molecules of *EPHB4* pathway in K562 cells with treatment with control or vandetanib for 48 h. Each column represents one sample, and each mRNA was depicted by one row, where red denotes an increase in mRNA expression and green denotes a decrease in mRNA expression as compared with the other group. (F) Protein level of EPHB4 in K562 cells treated with vandetanib (0, 0.78, 1.56 and 3.12 μm) for 48 h was examined by western blot assay. *P*‐values were generated by one‐way ANOVA using the Tukey test for multiple comparisons. *P < 0.05, **P < 0.01 versus the untreated control group. (G) Protein level of EPHB4 in K562 cells treated with different concentration of vandetanib and MG‐132 for 48 h. *P*‐values were generated by one‐way ANOVA using the Tukey test for multiple comparisons. *P < 0.05, **P < 0.01. (H) The ubiquitin of EPHB4 induced by vandetanib. (I) Expression levels of PI3K‐P110α, PI3K‐P110β, PI3K‐P110γ, p‐PI3K‐P85/p55 and PI3K‐P85, and the phosphorylation of AKT and MTOR in cell lysates of K562 cells treated with vandetanib for 48 h. (J) The phosphorylation of MEK1/2, ERK1/2 and P38 in cell lysates of K562 cells treated with vandetanib for 48 h. All the results were quantified by densitometry analysis of the bands and normalization to GAPDH. Data represent the means ± SEM (*n* = 3).

Genome Array assay followed by Gene‐Cloud of Biotechnology Information was performed to investigate the biological consequences of vandetanib treatment with an emphasis on changes in EPHB4 signaling pathways. Unsupervised hierarchical clustering showed that control (DMSO)‐ and vandetanib‐treated K562 cells had distinct patterns of mRNA expression (Fig. S7). A group of differentially expressed mRNAs was obtained by gene ontology (GO) enrichment and decreased pathway analysis, respectively. Vandetanib treatment led to significant changes in the mRNA expression levels of apoptosis‐related signal molecules such as *BCL‐2*, *MCL‐1*, *BAK*, *BAD* and *BAX* and a variety of signaling molecules such as *P53*, *PTEN* and *RAC1* (Fig. 3A). Also, EPHB4 knockdown downregulated the protein level of BCL‐2 and MCL‐1 and upregulated the protein level of BAX shown in Fig. S8A. Western blot analysis showed that P53 and PTEN were upregulated, and RAC1 was downregulated (Fig. 3B), which was consistent with Genome Array results. In addition, the protein expression levels of anti‐apoptotic members in BCL‐2 family such as BCL‐2 and MCL‐1 were downregulated, while those of pro‐apoptotic members, such as BAD, BAX and BAK, were upregulated (Fig. 3B). Moreover, vandetanib regulated the main protein levels of apoptotic‐related members (BCL‐2, MCL‐1 and BAX) in a time‐dependent manner (Fig. S8B). Thus, we went on to evaluate the effect of vandetanib on cell apoptosis. As shown in Fig. 3C, the population of Annexin V‐FITC‐positive cells (in early and late apoptotic stage) was significantly increased in vandetanib treatment group (15.95% for 0.78 μμ, 22.25% for 1.56 μμ and 52.03% for 3.12 μμ) compared with untreated control (3.53%) in K562 cells. Also, the apoptotic induction by vandetanib was more dramatic in K562 cells than in H9 cells (Fig. 3D). Moreover, vandetanib treatment could also induce CML MEG‐01 cell apoptosis in a dose‐dependent manner (6.60% for control, 15.52% for 0.30 μμ, 24.58% for 0.60 μμ and 42.23% for 1.20 μμ) which is shown in Fig. S9.

As the evidence that EPHB4 could be considered as a therapeutic target of vandetanib, HEK293 cell line transfected with EPHB4 (Fig. S10A) was further evaluated. Figure S10B shows that vandetanib was more sensitive to EPHB4 transfected HEK293 cells which express a higher level of EPHB4 compared to wild‐type HEK293 cells. In addition, the apoptotic induction by vandetanib was more dramatic in EPHB4 transfected HEK293 cells than in wild‐type HEK293 cells (Fig. S10C,D). These results were consistent with the effect of vandetanib on K562 cells.

It is interesting to note that vandetanib could downregulate the EPHB4 expression at both mRNA (Fig. 3E) and protein level (Fig. 3F) in K562 cells. In addition, vandetanib could also decrease the phosphorylation of EPHB4 in K562 cells (Fig. 3F). Moreover, vandetanib treatment dramatically downregulates the protein level of EPHB4 and the phosphorylation of EPHB4 in MEG‐01 cells (Fig. S11). Figure 3G shows that MG‐132 treatment rapidly and dramatically increased EPHB4 protein expression in these cells. In addition, EPHB4 ubiquitylation is directly enhanced by vandetanib treatment (Fig. 3H). These data indicated that the downregulation in the protein expression level of EPHB4 was, at least in part, mediated by ubiquitin‐proteasome pathway.

Ephrin type‐B receptor 4 knockdown downregulated the main members of PI3K/AKT and MAPK signaling (such as PI3K P85, p‐AKT, p‐MTOR and p‐ERK1/2) shown in Fig. S12A. In addition, several important components in PI3K/AKT and MAPK/ERK signaling pathways were also investigated by western blot following treatment with vandetanib (Fig. 3I,J). As shown in Fig. 3I, the protein levels of p‐PI3K‐P85/P55 and PI3K‐P85/P55, two main subunits of PI3K, were decreased in K562 cells following vandetanib treatment. The phosphorylation of AKT and MTOR was also reduced by vandetanib treatment. Vandetanib treatment also led to decreased phosphorylation of MEK1/2 and ERK1/2 and increased phosphorylation of P38 in K562 cells (Fig. 3J) in a dose‐dependent manner. Moreover, vandetanib regulated the main protein levels of EPHB4 and main downstream signaling members (PI3K P85, p‐AKT, p‐MTOR, p‐ERK1/2) in a time‐dependent manner (Fig. S12B).

### 3.5 Vandetanib bound EPHB4 motifs

Molecular dynamics simulations were then used to determine the binding site of vandetanib on EPHB4 and its mode of binding. As shown in Fig. S13A,B, the two simulation systems (EPHB4 and EPHB4‐vandetanib) were stable during the entire dynamics simulation process, with the temperature being constant at approximately 298 K and with no abnormal changes in energy. Meanwhile, the energy of the EPHB4‐vandetanib system was lower than that of the EPHB4 system (Fig. S13B), and the root mean square deviation (RMSD) value of the main chain atoms of EPHB4 in the complex (EPHB4‐vandetanib) was lower (around 2.7 Å) than that of EPHB4 alone (around 3.0 Å; Fig. S13C). In addition, compared with that of EPHB4 alone, the residues of the ligand‐binding region in the complex (EPHB4‐vandetanib) fluctuated much more dramatically and the other regions were less affected by ligands (Fig. S13D). The above data indicate that the system was more stable after binding to vandetanib.

The MM/PBSA method [35, 36] was also used to further calculate the binding free energy of the complex system. As shown in Table S9, the Van der Waal's energy (∆Evdw) had a major positive impact on the ligand binding and was the main driving force in the binding of vandetanib to EPHB4.

**Fig. 4:**
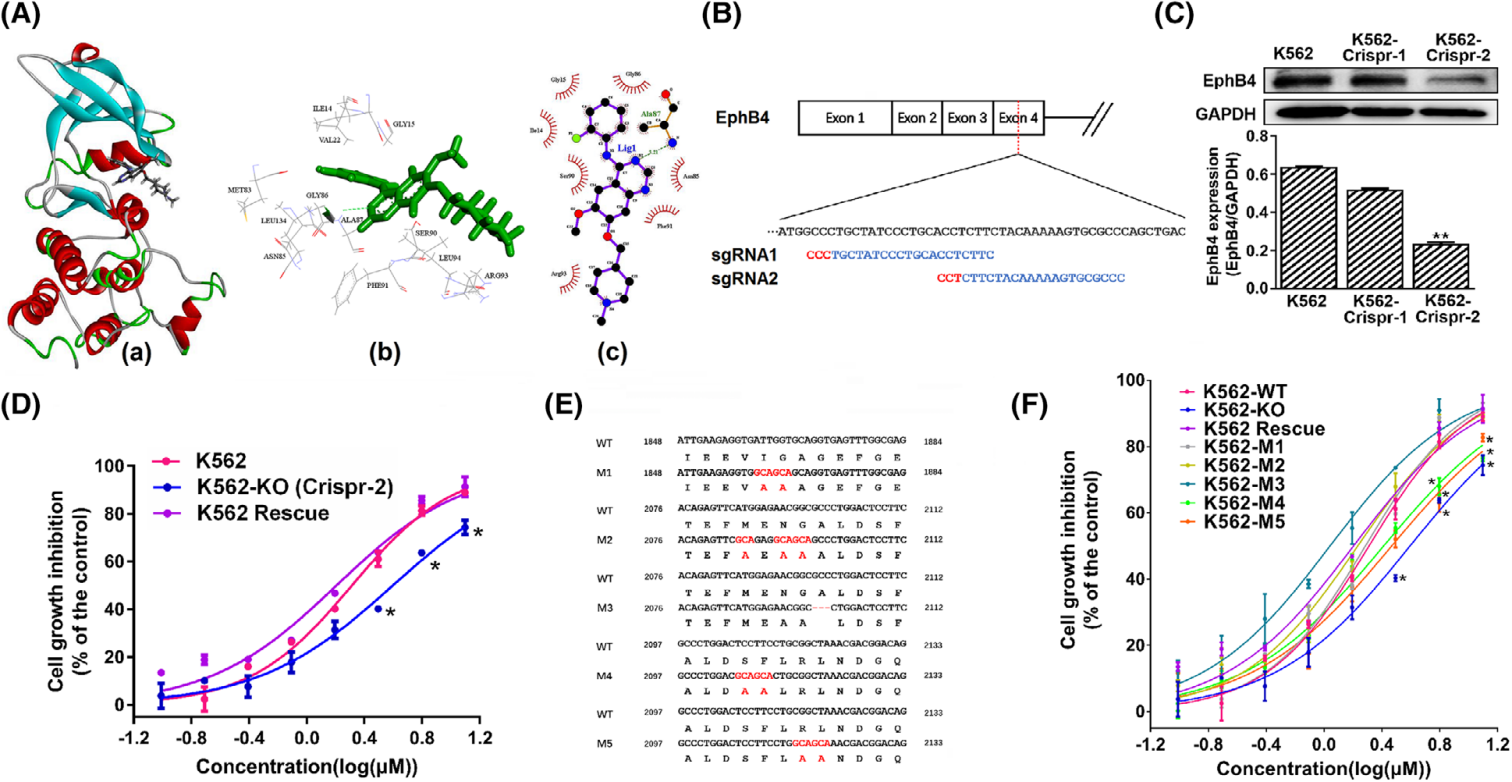
Vandetanib bound ephrin type‐B receptor 4 (EPHB4) motifs. (A) The structure graphs of the vandetanib and EPHB4 with the lowest energy from molecular dynamics (MD) track in the last 20 ns equilibrium by using the autodock 4.2. (a) Three‐dimensional structure graph; (b) binding region; (c) plane schematic of interaction between the receptor and ligand. (B) Genomic position of sgRNAs and PAMs for *EPHB4* knock‐out line, PAM site was colored in red. (C) The protein expression of EPHB4 in K562, K562‐Crispr‐1 and K562‐Crispr‐2 cells determined by western blot assay. *P*‐values were generated by one‐way ANOVA using the Tukey test for multiple comparisons. **P < 0.01 versus the K562 group. (D) Vandetanib sensitivity analysis on wild‐type, EPHB4 knock‐out and EPHB4 rescue cell lines after treatment for 48 h. *P*‐values were generated by one‐way ANOVA using the Tukey test for multiple comparisons. *P < 0.05 versus the K562 group. (E) Five point‐mutation (M1–M5) and their corresponding wild‐type (WT) sequences. The point‐mutations of EPHB4: M1, I14A, G15A; M2, M83A, N85A, G86A; M3, A87Del; M4, S90A, F91A; M5, R93A, L94A. (F) Vandetanib sensitivity analysis on mutation lines after treatment for 48 h. The curves of each mutation lines demonstrate the relative cell survival rate on different drug concentration. *P*‐values were generated by one‐way ANOVA using the Tukey test for multiple comparisons. *P < 0.05 versus the K562‐WT group. Data represent the means ± SEM (*n* = 3).

To further explore the binding mode of the active vandetanib‐binding pockets in EPHB4 protein, the interaction between vandetanib and EPHB4 protein residues was analyzed in detail. A structure with the lowest energy from MD track in the last 20 ns equilibrium was selected to analyze the interactions between the ligand in its binding pocket and the related residues by using the autodock 4.2 (Fig. 4A). Vandetanib bound to an aniline ring and a piperidine ring by a quinazoline parent nucleus. The F and Br substitutions of the aniline ring inserted into the inner grooves of the protein cavities showed a maximum overlap between the hydrophobic regions of the EPHB4 protein and the plane of the EPHB4 parent nucleus to ensure the stability of vandetanib inside the EPHB4 receptor. To further stabilize the interaction between vandetanib and EPHB4, the methoxy group present on the parent nucleus extended into the outer space on the edge of the hydrophobic pocket. The anilino group of the piperidine ring was close to an α‐helix (residue 41–53). Finally, vandetanib interacted stably with a hydrophobic pocket in EPHB4 composed of 12 residues including Isoleucine14, Glycine15, Valine 22, Methionine83, Asparagine85, Glycien86, Alanine87, Serine90, Phenylalanine91, Arginine93, Leucine94 and Leucine134.

Based on the MD simulations, the above‐mentioned 12 residues likely function as the major binding site for vandetanib. To test this hypothesis, several K562 sublines with selective mutation in these residues were generated. SgRNAs for EPHB4 knockout were designed at exon 4 according to protein domain conservation as shown in Fig. 4B. According to available online *in silico* analysis, *EPHB4*‐Crispr‐1 showed strong off‐target prediction, whereas *EPHB4*‐Crispr‐2 was expected to be good as the gRNA targeting EPHB4. It was also in line with the western blotting analysis that only *EPHB4*‐Crispr‐2 plasmid transfection significantly downregulated the protein expression of EPHB4 in K562 cells (Fig. 4C). Moreover, *EPHB4*‐Crispr‐1 transfection had no obvious effect on inhibitory effect of vandetanib compared with wild‐type control (Fig. S14). Therefore, *EPHB4*‐Crispr‐2 was selected to continue all the subsequent experiments. The positive K562 knockout cells were confirmed to have lower expression of EPHB4 (via qPCR and western blot; Fig. S15) and significantly decreased sensitivity to vandetanib compared with wild‐type control (Fig. 4D). Similar results were obtained in siRNA knockdown of EPHB4 (Fig. S16). In addition, the growth defects could be fully rescued by transfection of EPHB4 (Fig. S15 and Fig. 4D). The K562 EPHB4‐KO cells were subsequently used to generate five sublines with defined mutations in the 12 aa residues. The five mutants M1 (I14A, G15A), M2 (M83A, N85A, G86A), M3 (A87Del), M4 (S90A, F91A) and M5 (R93A, L94A) cover 10 out of the 12 putative hotspots as shown in Fig. 4E and were confirmed by sequencing (Fig. S17).

The five mutant sublines were treated with various concentrations of vandetanib for 48 h. As shown in Fig. 4F and Table S10, the M5 and M4 mutants showed significantly higher cell viability compared to wild‐type K562 cells. At the high‐concentration range of vandetanib, the M5 mutant showed a drug response curve that was similar to that of EPHB4‐knockout subline. Similar results were shown for M4 mutants. On the other hand, the other three mutants were comparable to wild‐type K562 cells in drug response, suggesting that A93&L94 and S90&91 may function as a core docking site for vandetanib.

### 3.6 The inhibitory action of vandetanib on K562 tumor *in vivo*

**Fig. 5:**
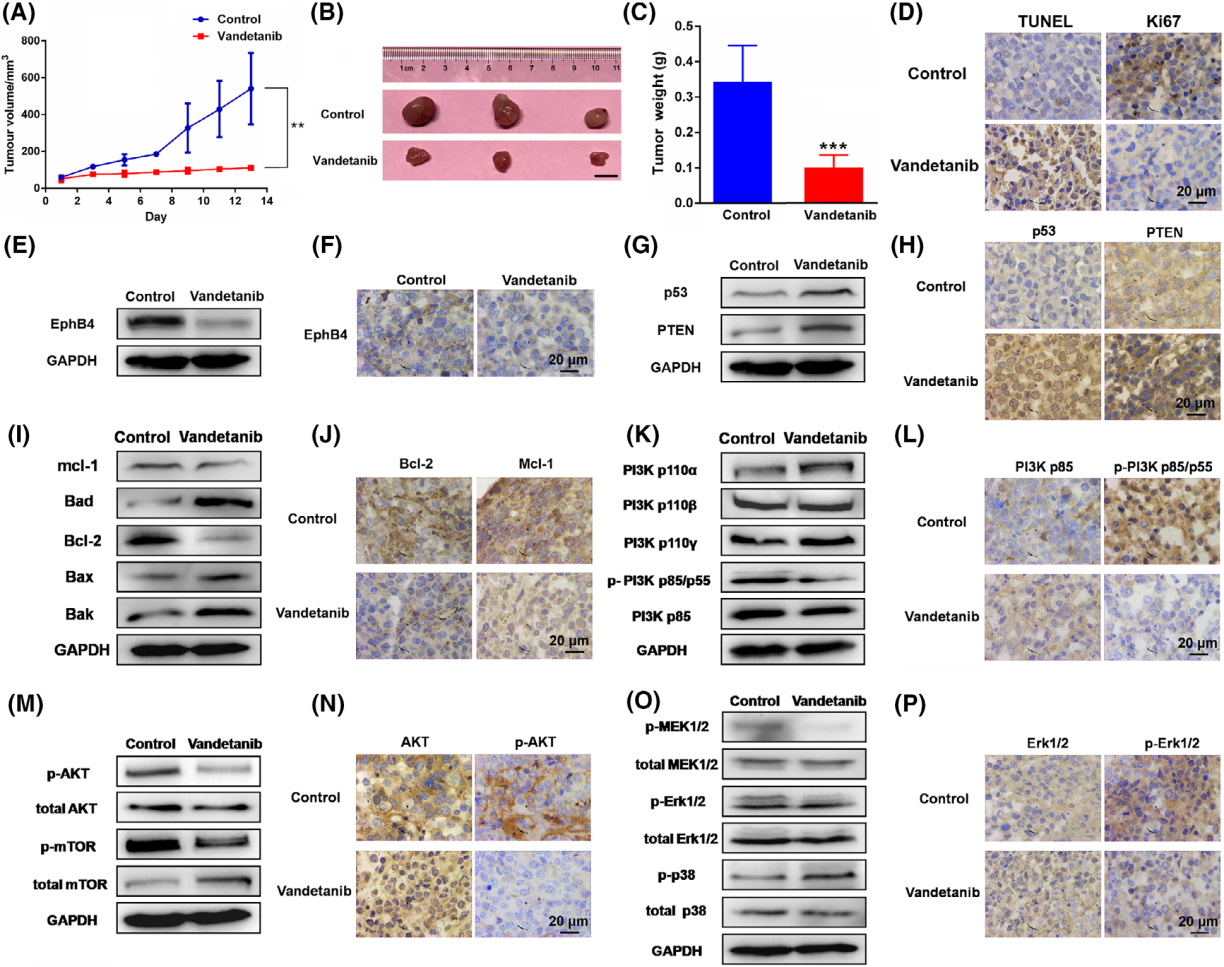
The inhibitory action of vandetanib on K562 tumor *in vivo*. (A) Tumor sizes were plotted as tumor volumes at different time point. Vandetanib dose was 40 mg·kg^−1^. *P*‐values were generated by Student's *t*‐test. **P < 0.01 versus control group. (B) The representative image of the tumor with different treatment at day 14. Scale bar, 1 cm. (C) The tumor weight of each group at day 14. *P*‐values were generated by Student's *t*‐test. ***P < 0.001 versus control group. (D) The immunohistochemical analysis of TUNEL and Ki67 in K562 tumor tissues. Scale bar, 20 μm. (E) the protein amount of ephrin type‐B receptor 4 (EPHB4) in K562 tumor tissues. (F) The immunohistochemical analysis of EPHB4 in K562 tumor tissues. Scale bar, 20 μm. (G) The protein amount of P53 and PTEN in K562 tumor tissues. (H) The immunohistochemical analysis of P53 and PTEN in K562 tumor tissues. Scale bar, 20 μm. (I) The protein amount of apoptosis related protein expression in K562 tumor tissues. (J) The immunohistochemical analysis of BCL‐2, MCL‐1 in K562 tumor tissues. Scale bar, 20 μm. (K) The protein amount of PI3K‐P110α, PI3K‐P110β, PI3K‐P110γ, p‐PI3K‐P85/p55 and PI3K‐P85 in K562 tumor tissues. (L) The immunohistochemical analysis of PI3K P85 and p‐PI3K P85/p55 in K562 tumor tissues. Scale bar, 20 μm. (M) The protein amount of AKT, p‐AKT, MTOR and p‐MTOR in K562 tumor tissues. (N) The immunohistochemical analysis of AKT and p‐AKT in K562 tumor tissues. Scale bar, 20 μm. (O) The protein amount of MEK1/2, p‐MEK1/2, ERK1/2, p‐ERK1/2, P38 and p‐P38 in K562 tumor tissues. (P) The immunohistochemical analysis of ERK1/2 and p‐ERK1/2 in K562 tumor tissues. Scale bar, 20 μm. Data represent the means ± SEM (*n* = 3).

Figure 5A–C shows the *in vivo* antitumor activity of vandetanib at a dosage of 40 mg·kg^−1^. Vandetanib showed remarkable inhibition against wild‐type K562 tumor growth. To evaluate the *in vivo* effect of vandetanib on the proliferation and apoptosis of tumor cells, the tumor tissues were subjected to TUNEL and Ki67 cell proliferation assays. As shown in Fig. 5D, vandetanib treatment led to a significant increase in the number of TUNEL‐positive cells and a decrease in the number of Ki67‐positive cells, indicating that vandetanib could induce cell apoptosis and inhibit cell proliferation *in vivo*.

### 3.7 Effect of vandetanib on EPHB4 signaling *in vivo*

The protein expression of EPHB4 and its related molecules *in vivo* was investigated by immunohistochemistry and western blot which was quantified by densitometry analysis (Fig. S18). The results showed that EPHB4 expression was significantly decreased by vandetanib treatment (Fig. 5E,F), while the expression of the related molecules P53 and PTEN was upregulated (Fig. 5G,H). Moreover, vandetanib treatment could decrease the protein expression of MCL‐1 and BCL‐2 and increase the expression of BAD, BAX and BAK in K562 tumor tissues (Fig. 5I), which was consistent with the regulation of apoptosis‐related proteins in cancer cells *in vitro* (Fig. 3B). Furthermore, immunohistochemistry showed that the expression levels of BCL‐2 and MCL‐1, two major cell apoptosis‐related molecules, were decreased by vandetanib in K562 tumor tissues (Fig. 5J). The effect of vandetanib on some important components in the PI3K/AKT and MAPK/ERK signaling pathways was also investigated by western blot and immunohistochemistry. Treatment with vandetanib significantly decreased the levels of p‐PI3K P85/P55 and the phosphorylation of AKT, MTOR, MEK1/2 and ERK1/2 and increased the phosphorylation of P38 in K562 tumor tissues (Fig. 5K,M,O). These western results were confirmed by immunohistochemistry in K562 tumor tissues (Fig. 5L,N,P).

### 3.8 The synergetic action of vandetanib on imatinib‐resistant K562 tumor

**Fig. 6:**
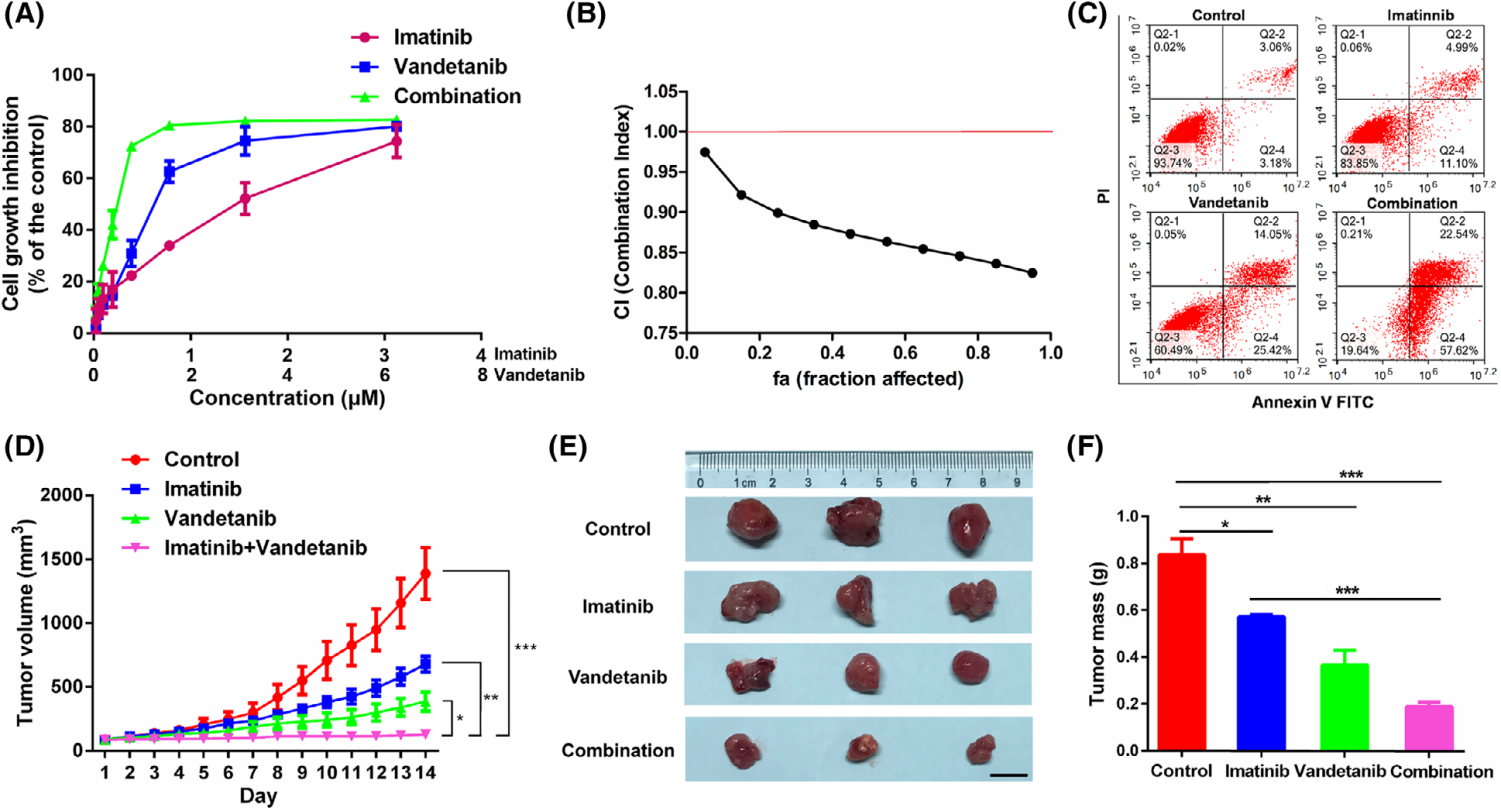
The synergetic action of vandetanib on imatinib‐resistant K562 tumor *in vitro* and *in vivo*. (A) Dose–response study of a fixed ratio combination of imatinib (0.0–3.12 μm) and vandetanib (0.0–6.25 μm) for 48 h against K562 cells. (B) Fa‐CI plot in which fa and CI indicate fraction affected and combination index, respectively. CI < 1, CI = 1 and CI > 1 denote synergistic, additive and antagonistic interaction, respectively. (C) Effect of vandetanib alone, imatinib alone and combination for 48 h on cell apoptosis in imatinib‐resistant K562 cells. (D) Tumor sizes were plotted as tumor volumes at different time point in imatinib‐resistant K562 tumor model. Vandetanib dose was 40 mg·kg^−1^ and imatinib dose was 100 mg·kg^−1^. *P*‐values were generated by one‐way ANOVA using the Tukey test for multiple comparisons. *P < 0.05, **P < 0.01, ***P < 0.001 versus control group. (E) The representative image of the tumor with different treatment in imatinib‐resistant K562 tumor model at day 14. Scale bar, 1 cm. (F) The tumor weight of each group at day 14 in imatinib‐resistant K562 tumor model. Data represent the means ± SEM (*n* = 3). *P*‐values were generated by one‐way ANOVA using the Tukey test for multiple comparisons. *P < 0.05, **P < 0.01, ***P < 0.001 versus control group.

After demonstrating the direct inhibitory effect of vandetanib on wild‐type K562 cells *in vitro* and *in vivo*, we further examined whether combination with vandetanib could sensitize imatinib‐resistant K562 cells to imatinib treatment. Imatinib‐resistant K562 cancer cells were developed via treating with incremental concentrations of imatinib [37]. As shown in Fig. 6A,B, combination of vandetanib and imatinib resulted in an enhanced and synergistic growth inhibition against imatinib‐resistant K562 cells compared to the effects from vandetanib or imatinib alone. In addition, combination group (80.16%) was more effective in inducing cell apoptosis compared to treatment with vandetanib (39.47%) or imatinib (16.09%) alone (Fig. 6C). Figure 6D–F shows the *in vivo* antitumor activity of the combination treatment in an imatinib‐resistant K562 xenograft model at an imatinib dosage of 100 mg·kg^−1^ and a vandetanib dose of 40 mg·kg^−1^, respectively. Imatinib alone showed minimal effect in inhibiting the tumor growth. Vandetanib was more effective than imatinib in inhibiting the tumor growth. The combination of the two drugs led to the best therapeutic outcome compared to the single treatment groups.

## 4 Discussion

Ephrin type‐B receptor 4 plays key roles in cancer progression, such as cell growth, survival, angiogenesis, migration, metastasis and so on [38]. EPHB4 has become important components of various cancer treatment strategies [10, 38]. Recent reports show that EPHB4 is overexpressed in K562 cell line [10, 18], and high expression of EPHB4 is associated with imatinib resistance [9, 19] and dasatinib resistance [17], suggesting that EPHB4 may represent a potential target for CML therapy. In this study, the role of EPHB4 in CML was evaluated. Our data showed that EPHB4 was highly expressed in CML, and knockdown of EPHB4 expression in K562 cells led to significant slow‐down of cell growth *in vitro* and the growth defects could be fully rescued by transfection with EPHB4. The above data strongly suggest that *EPHB4* is an oncogene in CML that plays an important role in both tumor cell growth and drug response to imatinib. Hence, we attempted to screen potential drugs that can exert anti‐CML activity via targeting EPHB4. In this study, we found that vandetanib possessed remarkable antiproliferative effects on CML cells as an EPHB4 inhibitor.

To identify an EPHB4 inhibitor with a potential of rapid translation into clinic, we screened a pool of clinically approved compounds using hematologic cancer cells expressing different levels of EPHB4. K562 cells that express a high level of EPHB4 were most sensitive to vandetanib, suggesting that vandetanib might inhibit the cell growth via targeting EPHB4. Furthermore, the mRNA expression of *EPHB4* in blood samples was much higher in K562 model than in H9 model, and treatment with vandetanib led to a downregulation in the mRNA expression level of *EPHB4 in vivo*. More importantly, vandetanib showed remarkable inhibition against wild‐type K562 tumor growth *in vivo*. All above data suggested that vandetanib inhibits K562 tumor growth *in vitro* and *in vivo*.

We next went on to verify whether EPHB4 could be a therapeutic target for vandetanib. The data of SPR and AC showed that vandetanib could bind to both the extracellular functional site and the intracellular domain (the active domain of tyrosine kinase) of EPHB4 with a higher affinity for the latter, suggesting that vandetanib could affect the kinase activity of EPHB4. Accordingly, the inhibitory effects on the EPHB4 kinase activity were evaluated. Vandetanib altered EPHB4 kinase activity in a dose‐dependent manner and the effect of vandetanib was much more dramatic than NVP‐BHG712 (a small molecule EPHB4 kinase‐specific inhibitor). These data indicate that vandetanib could bind to the intracellular domain of EPHB4 and inhibit its kinase activity. In addition, our data suggested that vandetanib inhibited the growth of BaF3‐EPHB4 cells via targeting EPHB4 and with a significantly higher selectivity over NVP‐BHG712. These data validate that vandetanib could be an EPHB4 inhibitor.

Following establishment of EPHB4 as the molecular target of vandetanib, we went on to perform Genome Array assay to systematically investigate the biological consequences of vandetanib treatment with an emphasis on changes in EPHB4 signaling pathways. It is interesting to note that vandetanib could downregulate the protein level of EPHB4 in CML cells, which was mediated by transcriptional repression and ubiquitin‐proteasome pathway. Similar results were obtained *in vivo*. In addition, vandetanib could also downregulate the phosphorylation of EPHB4 in CML cells. In consistent with an important role of EPHB4 in regulation of cell apoptosis by modulation of apoptosis‐related signaling molecules like MCL‐1 [17, 39], vandetanib treatment led to significant upregulation in the expression of P53, PTEN, BAD, BAX and BAK and downregulation in the expression of RAC1, BCL‐2 and MCL‐1 at both mRNA level and protein level. Similar results were shown *in vivo*. As a consequence, vandetanib induced CML cell apoptosis in a dose‐dependent manner, which was more dramatic than in H9 cells. Also, vandetanib treatment led to a significant increase in the number of TUNEL‐positive cells in K562 tumor tissue, indicating vandetanib could induce cell apoptosis *in vivo*. These results indicate that vandetanib could induce CML cell apoptosis both *in vitro* and *in vivo*, which might be correlated with EPHB4.

PI3K/AKT and MAPK/ERK are two intracellular pathways that play an important role in cell growth [40, 41, 42]. Studies showed that PI3K/AKT and MAPK/ERK signaling pathways could be regulated by EPHB4 [43, 44, 45]. Our data showed vandetanib could downregulate the protein levels of p‐PI3K‐P85/P55 and PI3K‐P85/P55, two main subunits of PI3K and the phosphorylation of AKT and MTOR. In addition, vandetanib treatment also led to decreased phosphorylation of MEK1/2 and ERK1/2 and increased phosphorylation of P38 in K562 cells. Similar results were obtained in K562 tumor tissue. The above results suggest that vandetanib could regulate cell growth through modulating PI3K/AKT and MAPK/ERK signaling.

As the evidence that vandetanib could target EPHB4 and regulate the downstream signalings, MD simulations were used to investigate the binding mode and site of vandetanib on EPHB4. Our data showed the structure of vandetanib‐bound EPHB4 was more stable than that of EPHB4 alone, and Van der Waal's energy was the main driving force in the binding of vandetanib to EPHB4. Furthermore, the residues (Serine90, Phenylalanine91, Arginine93 and Leucine94) likely function as the core binding sites for vandetanib. Our data so far strongly support the notion that vandetanib is an EPHB4‐potent inhibitor with significantly improved selectivity over NVP‐BHG712, the best‐known inhibitor of EPHB4. Our structural study may also shed light on future development of further improved EPHB4 inhibitors.

Furthermore, transfection of *EPHB4* siRNA led to sensitization to imatinib, and transfection of EPHB4 could fully rescue the growth defect of K562 cells which were sensitive to imatinib. This indicated that inhibition of EPHB4 could sensitize K562 cells to imatinib, so we supposed that vandetanib as an EPHB4 inhibitor could increase the sensitivity of imatinib‐resistant K562 cells. Accordingly, the combination of vandetanib and imatinib led to enhanced and synergistic growth inhibition against imatinib‐resistant K562 cells *in vitro* and *in vivo*. These results indicate that vandetanib sensitized imatinib‐resistant K562 cells to imatinib.

## 5 Conclusions

Here, our study has established vandetanib as a potent inhibitor of EPHB4. Inhibition of EPHB4 by vandetanib drives growth arrest and promotes the sensitivity of imatinib‐resistant cells to imatinib in CML. Combined treatment with vandetanib and imatinib may provide a new therapeutic strategy to overcome acquired drug resistance in patients with CML. More importantly, the authors have provided positive data for the efficacy of vandetanib as a potential strategy against cancers with high‐EPHB4 expression.

## Conflict of interest

The authors declare no conflict of interest.

## Author contributions

YZhang and SL designed the project. WM wrote the paper. BW performed bioinformatics analysis. WM, MZ, ZG, XD, TY and XS performed the experiment. BD, YZhan and DZ analyzed data. YJ and YW read the paper.

## Peer review

The peer review history for this article is available at https://publons.com/publon/10.1002/1878-0261.13270.

## Supporting information


**Fig. S1.** pX459 v2.0 vector map. sgRNAs are inserted into BbsI restriction site.
**Fig. S2.** Effect of vandetanib (A), afatinib (B), lapatinib (C), sunitinib (D), sorafenib (E), and erlotinib (F) for 48 h on cell proliferation in K562, H9 and JLTRG cells was determined by MTT assay.
**Fig. S3.** Effect of vandetanib (A), afatinib (B), lapatinib (C) for 48 h on cell proliferation in FDC‐P1 cells was determined by MTT assay.
**Fig. S4.** Effect of vandetanib on cell growth in MEG‐01 cells in 24, 48 and 72 h.
**Fig. S5.** Binding affinity of afatinib (A) and lapatinib (B) with the intracellular domain and extracellular domain of EphB4 by SPR analysis.
**Fig. S6.** Effect of afatinib and lapatinib on EphB4 kinase activity.
**Fig. S7.** Hierarchical cluster analysis of the genetically different populations in K562 cells with treatment with control or vandetanib for 48 h.
**Fig. S8.** The effect of EphB4 and vandetanib in regulating the apoptotic related proteins.
**Fig. S9.** Effect of vandetanib treatment for 48 h on cell apoptosis in wild type MEG‐01 cells.
**Fig. S10.** Effect of EphB4 on HEK293 cell growth induced by vandetanib.
**Fig. S11.** Protein level of EphB4 and p‐EphB4 in MEG‐01 cells treated with vandetanib (0, 0.30, 0.60 and 1.20 μm) for 48 h were examined by western blot assay.
**Fig. S12.** The effect of EphB4 induced by vandetanib on downstream signaling members.
**Fig. S13.** The stability of molecular dynamics simulation system.
**Fig. S14.** Vandetanib sensitivity analysis on wild type, EphB4‐Crispr‐1 cell lines after treatment for 48 h.
**Fig. S15.** The mRNA and protein level of EphB4 on different cell lines.
**Fig. S16.** The role of EphB4 in the biological activity of vandetanib treatment.
**Fig. S17.** Sanger sequencing data of five point‐mutation lines (M1–M5) and the corresponding WT sequences.
**Fig. S18.** The results of western blot were quantified by densitometry analysis of the bands and normalization to GAPDH.
**Table S1.** The information about the antibody used in western blot assay.
**Table S2.** Primers sequences.
**Table S3.** Primers and oligos sequences.
**Table S4.** The K562 cell growth inhibition by compounds (10 μm) screened in this study.
**Table S5.** The IC_50_ of TKIs in K562 cells and H9 cells.
**Table S6.** The IC_50_ of vandetanib in K562 cells, MEG‐01 cells and H9 cells.
**Table S7.** The *K*_D_ values of TKIs interacting with EphB4 by SPR analysis.
**Table S8.** The IC_50_ of TKIs on EphB4 kinase activity by kinase assay.
**Table S9.** The predicted binding free energies and the individual energy components computed by MM/PBSA method (neglecting the configurational entropy, kcal·mol^−1^).
**Table S10.** The IC_50_ of vandetanib in K562 cell line and other mutant sublines.

## Data Availability

The authors declare that the data supporting the findings of this study are available within the paper and its supplementary information files. Raw data are available from the author upon reasonable request. Differentially expressed mRNAs comparing K562 cells treated with control and vandetanib were analyzed by microarray analysis, and the Publish repositories accession Code is GSE158518.
