# Supplementary material for: Vandetanib drives growth arrest and promotes sensitivity to imatinib in chronic myeloid leukemia by targeting ephrin type‐B receptor 4
Source: Mol Oncol. 2022 Jun 27;16(14):2747–65. doi: 10.1002/1878-0261.13270 (PMC9297786; doi:10.1002/1878-0261.13270)
Supplement: Supplementary file 1 — Fig. S1. pX459 v2.0 vector map. sgRNAs are inserted into BbsI restriction site. Fig. S2. Effect of vandetanib (A), afatinib (B), lapatinib (C), sunitinib (D), sorafenib (E), and erlotinib (F) for 48 h on cell proliferation in K562, H9 and JLTRG cells was determined by MTT assay. Fig. S3. Effect of vandetanib (A), afatinib (B), lapatinib (C) for 48 h on cell proliferation in FDC‐P1 cells was determined by MTT assay. Fig. S4. Effect of vandetanib on cell growth in MEG‐01 cells in 24, 48 and 72 h. Fig. S5. Binding affinity of afatinib (A) and lapatinib (B) with the intracellular domain and extracellular domain of EphB4 by SPR analysis. Fig. S6. Effect of afatinib and lapatinib on EphB4 kinase activity. Fig. S7. Hierarchical cluster analysis of the genetically different populations in K562 cells with treatment with control or vandetanib for 48 h. Fig. S8. The effect of EphB4 and vandetanib in regulating the apoptotic related proteins. Fig. S9. Effect of vandetanib treatment for 48 h on cell apoptosis in wild type MEG‐01 cells. Fig. S10. Effect of EphB4 on HEK293 cell growth induced by vandetanib. Fig. S11. Protein level of EphB4 and p‐EphB4 in MEG‐01 cells treated with vandetanib (0, 0.30, 0.60 and 1.20 μm) for 48 h were examined by western blot assay. Fig. S12. The effect of EphB4 induced by vandetanib on downstream signaling members. Fig. S13. The stability of molecular dynamics simulation system. Fig. S14. Vandetanib sensitivity analysis on wild type, EphB4‐Crispr‐1 cell lines after treatment for 48 h. Fig. S15. The mRNA and protein level of EphB4 on different cell lines. Fig. S16. The role of EphB4 in the biological activity of vandetanib treatment. Fig. S17. Sanger sequencing data of five point‐mutation lines (M1–M5) and the corresponding WT sequences. Fig. S18. The results of western blot were quantified by densitometry analysis of the bands and normalization to GAPDH. Table S1. The information about the antibody used in western blot assay. Table S2. Primers seque [file MOL2-16-2747-s001.docx]

**Supplementary Materials**

**1. Supplementary figures**

**2. Supplementary tables**

**1. Supplementary figures**


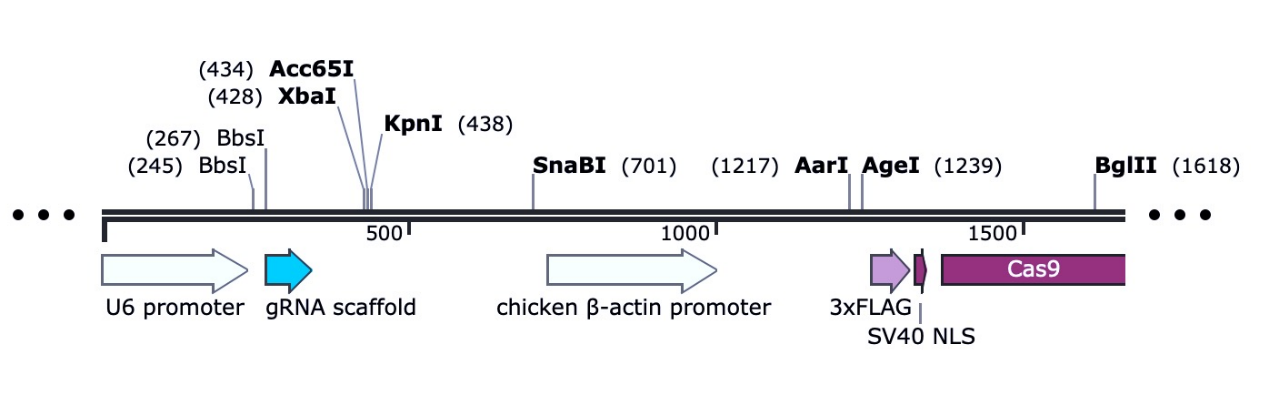


**Fig. S1** pX459 v2.0 vector map. sgRNAs are inserted into BbsI restriction site.


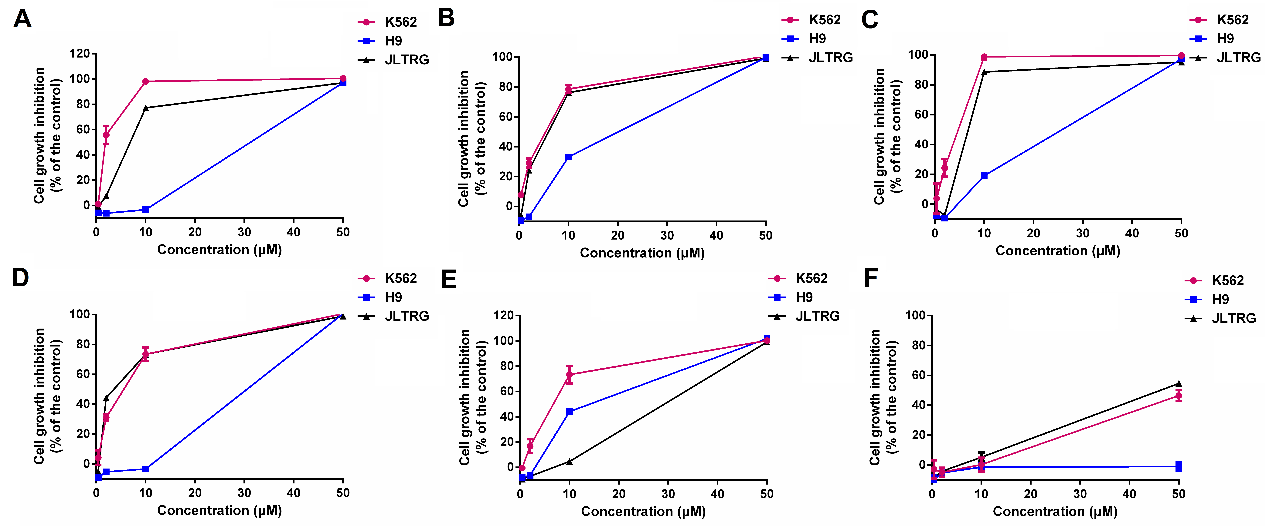


**Fig. S2** Effect of vandetanib **(A)**, afatinib **(B)**, lapatinib **(C)**, sunitinib **(D)**, sorafenib **(E)**, and erlotinib **(F)** for 48 h on cell proliferation in K562, H9 and JLTRG cells was determined by MTT assay. The values represent the average of three independent experiments. Data represents the means ± SEM (n=3).


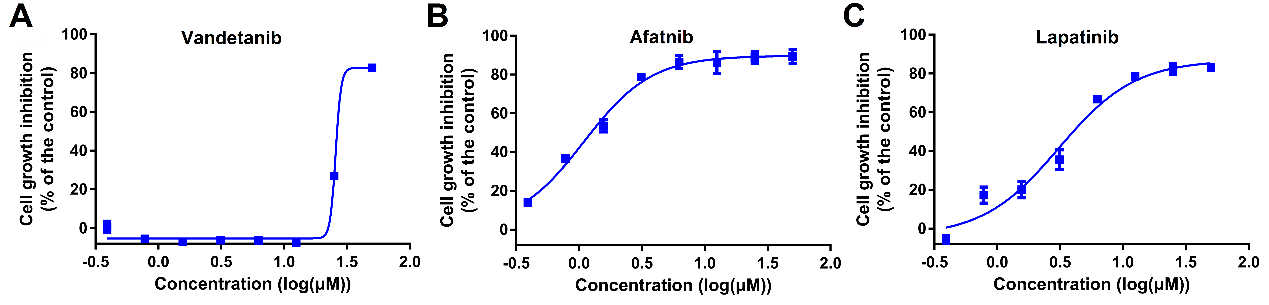


**Fig. S3** Effect of vandetanib **(A)**, afatinib **(B)**, lapatinib **(C)** for 48 h on cell proliferation in FDC-P1 cells was determined by MTT assay. The values represent the average of three independent experiments. Data represents the means ± SEM (n=3).


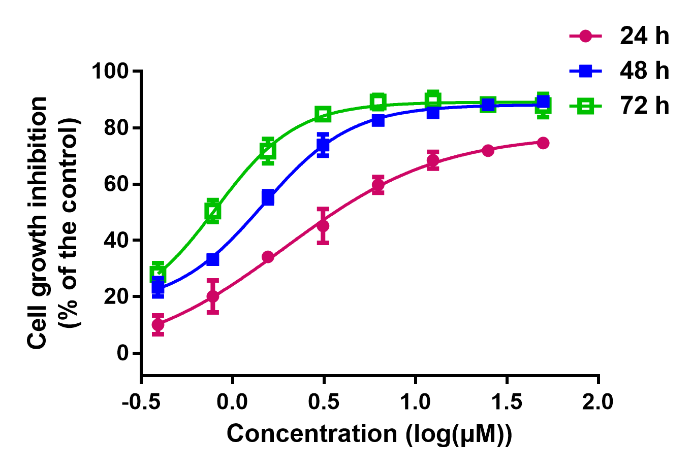


**Fig. S4** Effect of vandetanib on cell growth in MEG-01 cells in 24 h, 48 h and 72 h. The values represent the average of three independent experiments. Data represents the means ± SEM (n=3).


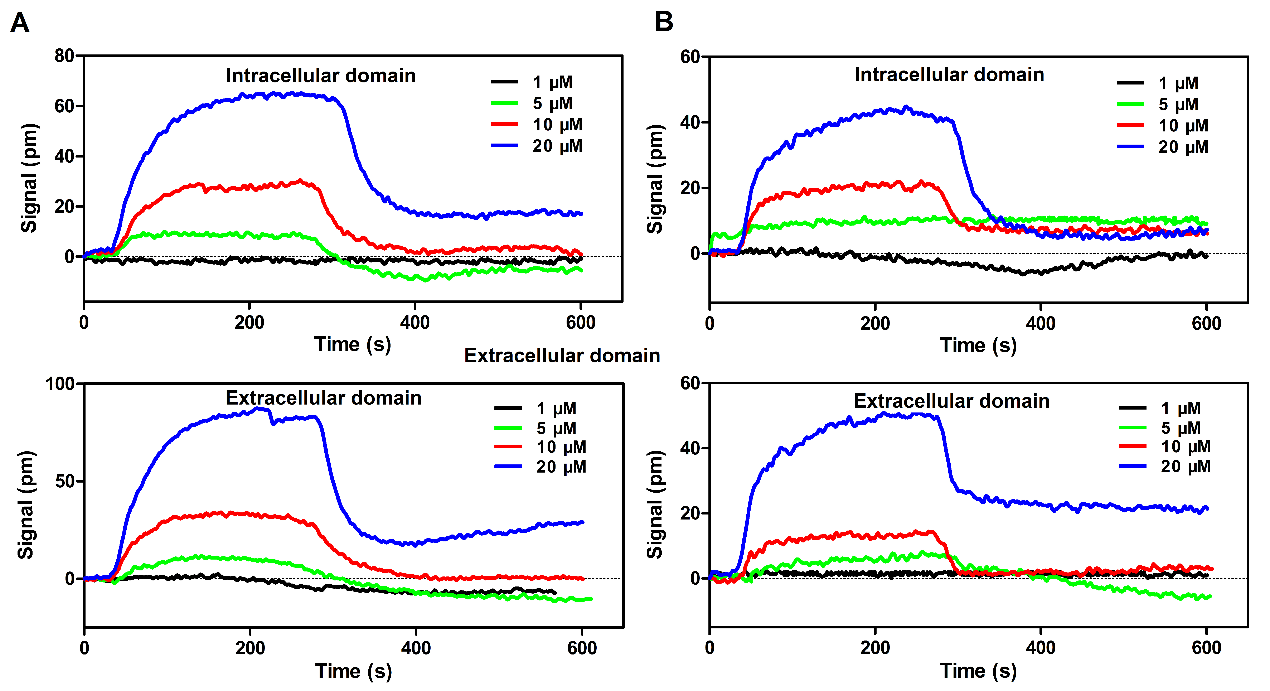


**Fig. S5** Binding affinity of afatinib **(A)** and lapatinib **(B)** with the intracellular domain and extracellular domain of EphB4 by SPR analysis.


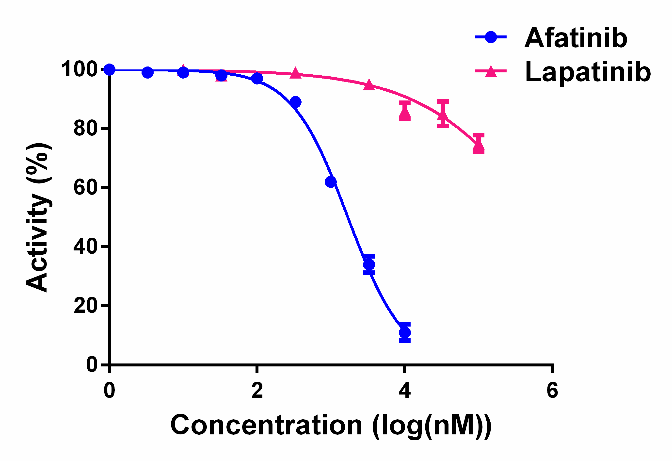


**Fig. S6** Effect of afatinib and lapatinib on EphB4 kinase activity.


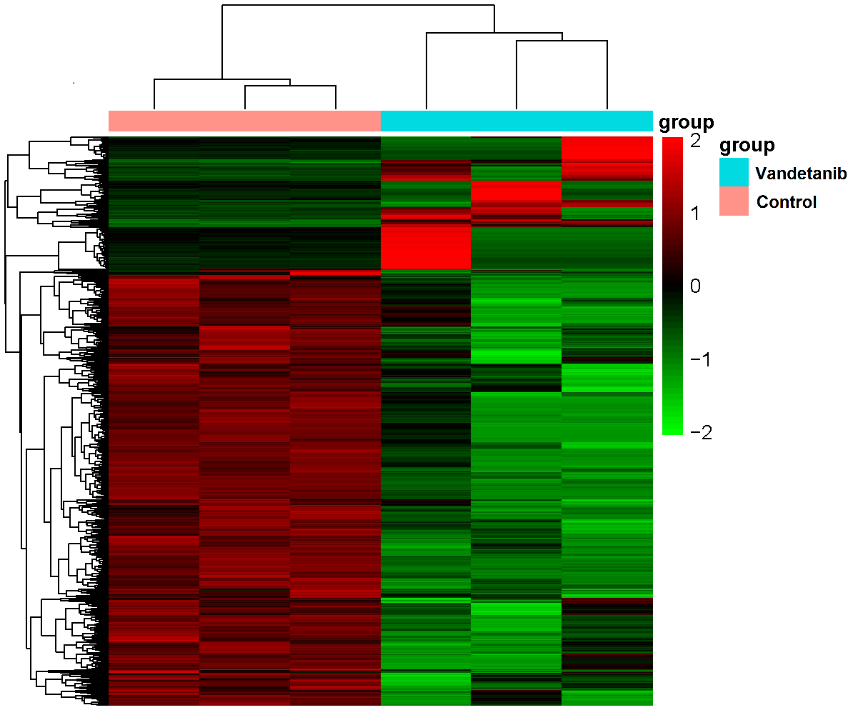


**Fig. S7** Hierarchical cluster analysis of the genetically different populations in K562 cells with treatment with control or vandetanib for 48 h. Each column represents one sample, and each gene was depicted by one row, where red denotes an increase in gene expression and green denotes a decrease in gene expression as compared with the other group.


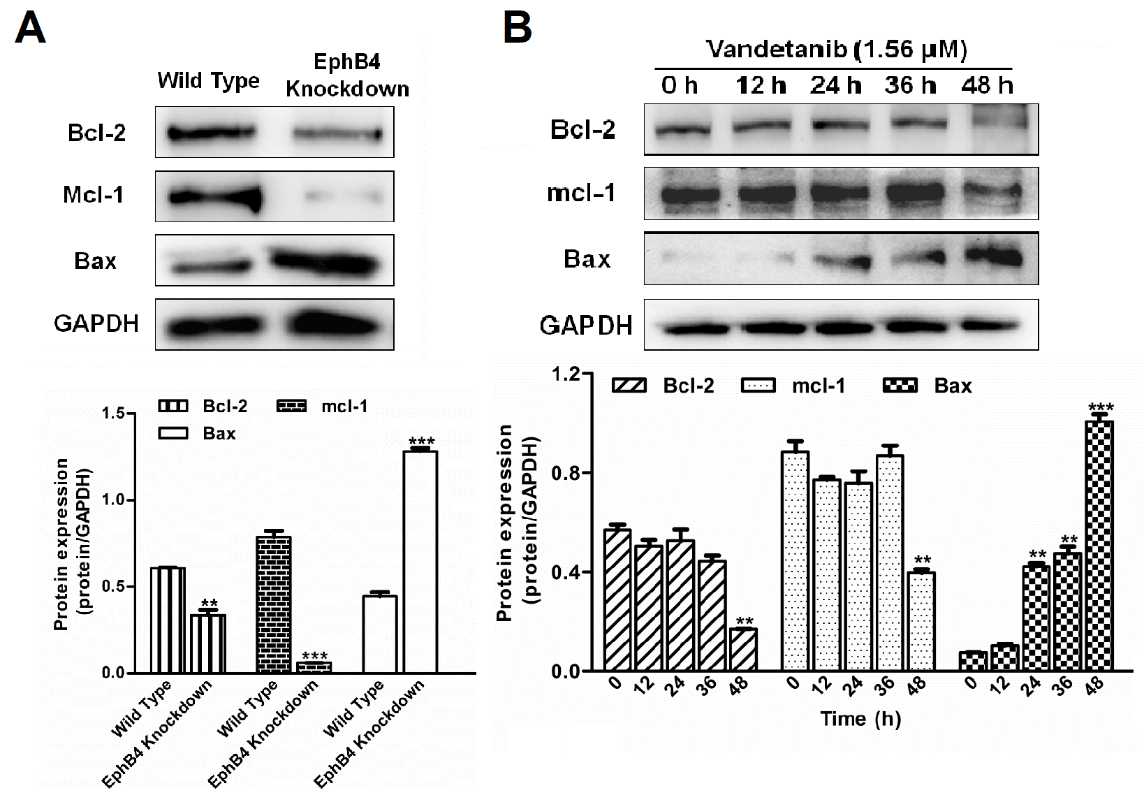


**Fig. S8** The effect of EphB4 and vandetanib in regulating the apoptotic related proteins. **(A)** The protein level of Bcl-2, Mcl-1, and Bax in wild type and EphB4 knockdown K562 cells. **p*< 0.05, ***p*< 0.01, ****p*< 0.001 versus the untreated control group. **(B)** Effect of vandetanib treatment on cell apoptosis related protein (Bcl-2, Mcl-1, and Bax) expression at different time. **p*< 0.05, ***p*< 0.01, ****p*< 0.001 versus the untreated control group. All the results were quantified by densitometry analysis of the bands and normalization to GAPDH. Data represents the means ± SEM (n=3).


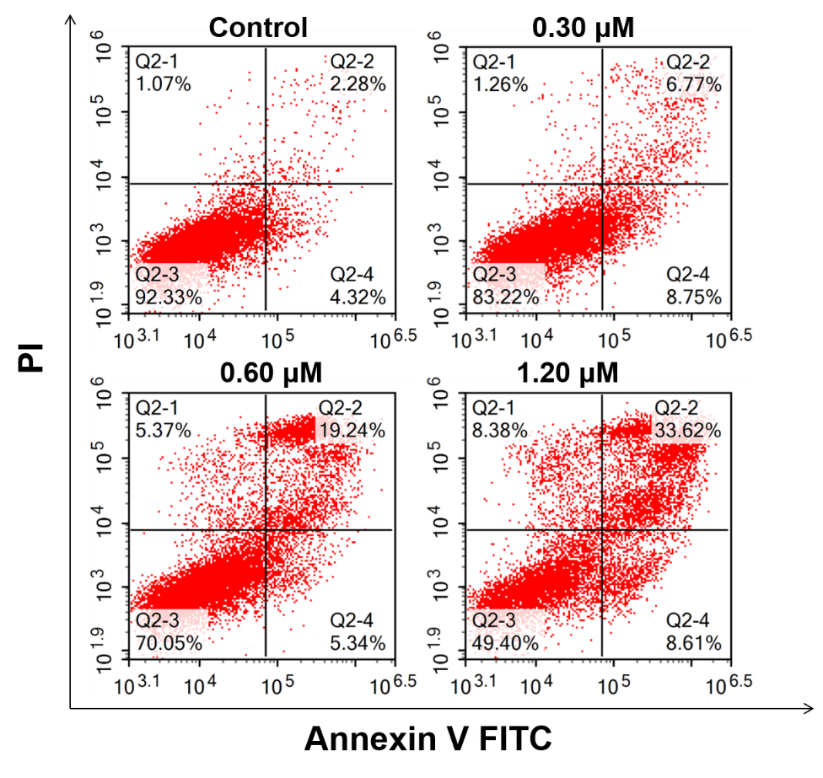


**Fig. S9** Effect of vandetanib treatment for 48 h on cell apoptosis in wild type MEG-01 cells.


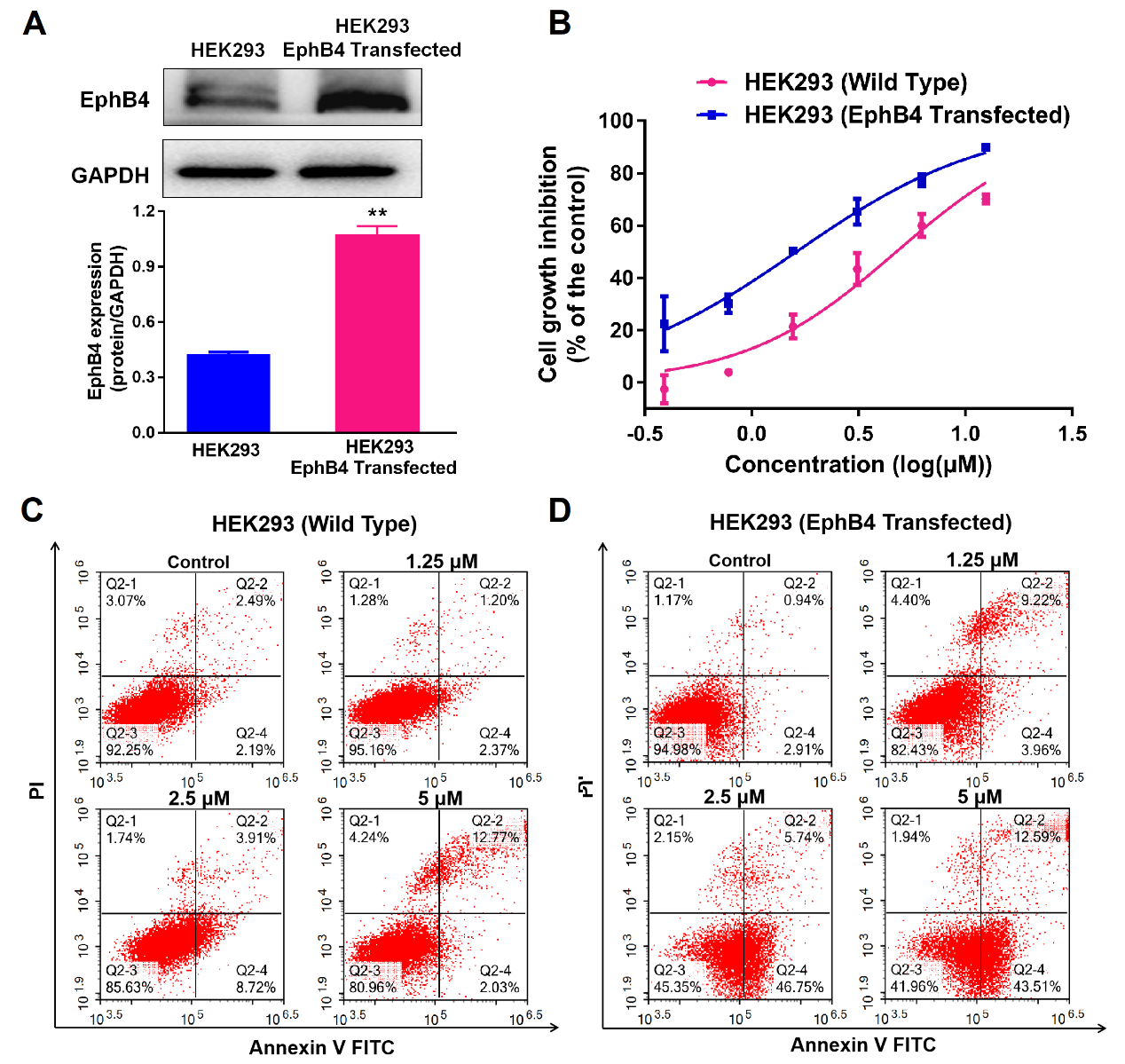


**Fig. S10** Effect of EphB4 on HEK293 cell growth induced by vandetanib. **(A)** The protein expression of EphB4 in wild type and EphB4 transfected HEK293 cells. **p*< 0.05, ***p*< 0.01 versus the untreated HEK293 group. **(B)** Effect of vandetanib for 48 h on cell proliferation in wild type and EphB4 transfected HEK293 cells was determined by MTT assay. **(C)** Effect of vandetanib treatment for 48 h on cell apoptosis in wild type HEK293 cells. **(D)** Effect of vandetanib treatment for 48 h on cell apoptosis in EphB4 transfected HEK293 cells. The values represent the average of three independent experiments. Data represents the means ± SEM (n=3).


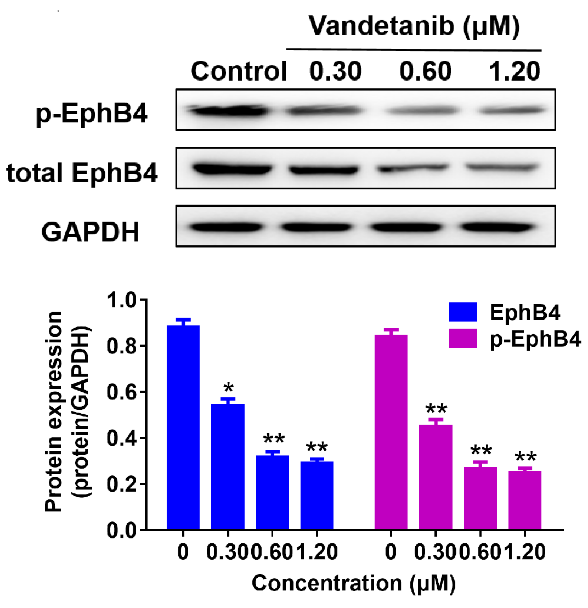


**Fig. S11** Protein level of EphB4 and p-EphB4 in MEG-01 cells treated with vandetanib (0, 0.30, 0.60 and 1.20 μM) for 48 h were examined by western blot assay. **p*< 0.05, ***p*< 0.01 versus the untreated control group.


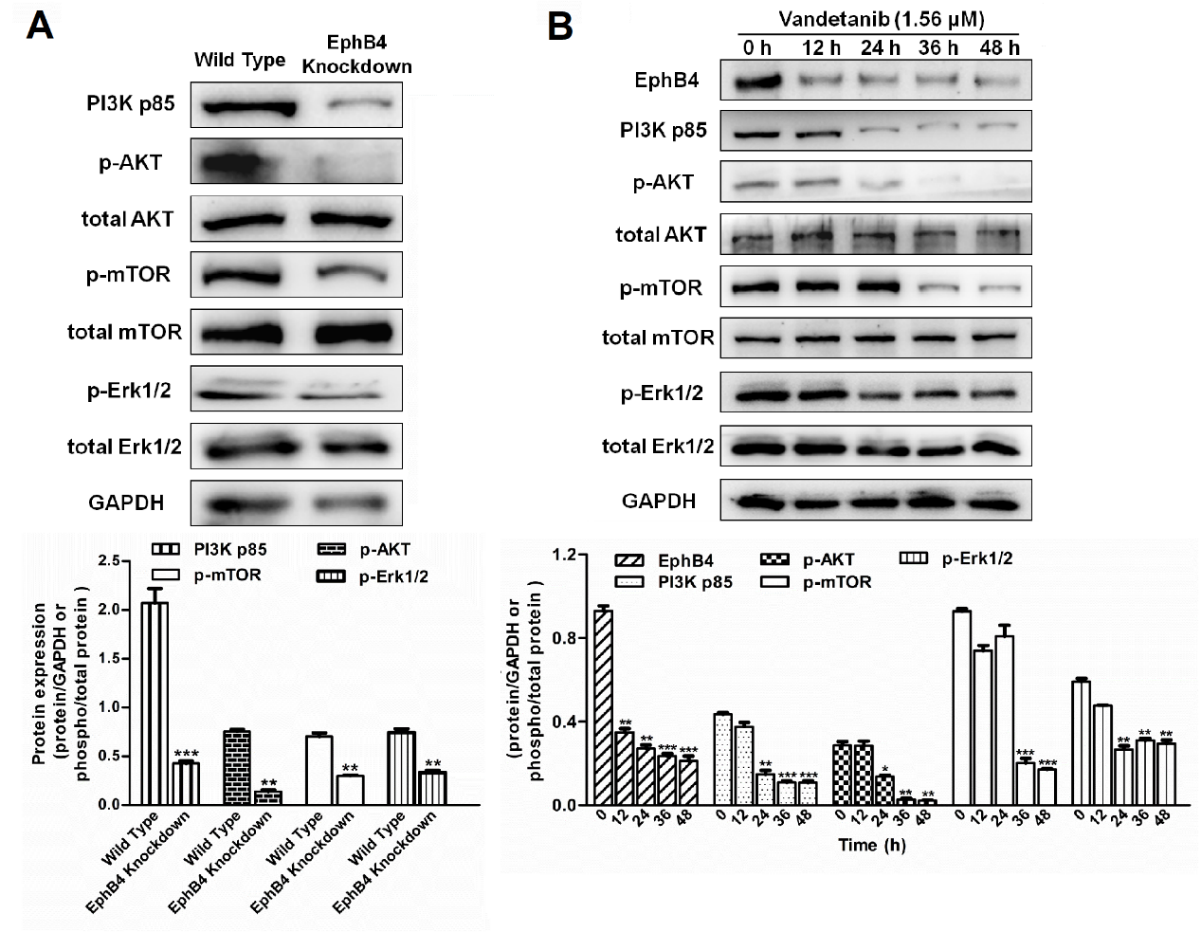


**Fig. S12** The effect of EphB4 induced by vandetanib on downstream signaling members. **(A)** The protein level of PI3K p85, p-AKT, AKT, p-mTOR, mTOR, p-Erk1/2 and Erk1/2 in wild type and EphB4 knockdown K562 cells. **p*< 0.05, ***p*< 0.01, ****p*< 0.001 versus the untreated control group. **(B)** Protein level of EphB4, p-PI3K-p85/p55, the phosphorylation of AKT, mTOR and Erk1/2 in cell lysates of K562 cells treated with vandetanib at different time. **p*< 0.05, ***p*< 0.01, ****p*< 0.001 versus the untreated control group. All the results were quantified by densitometry analysis of the bands and normalization to GAPDH. Data represents the means ± SEM (n=3).


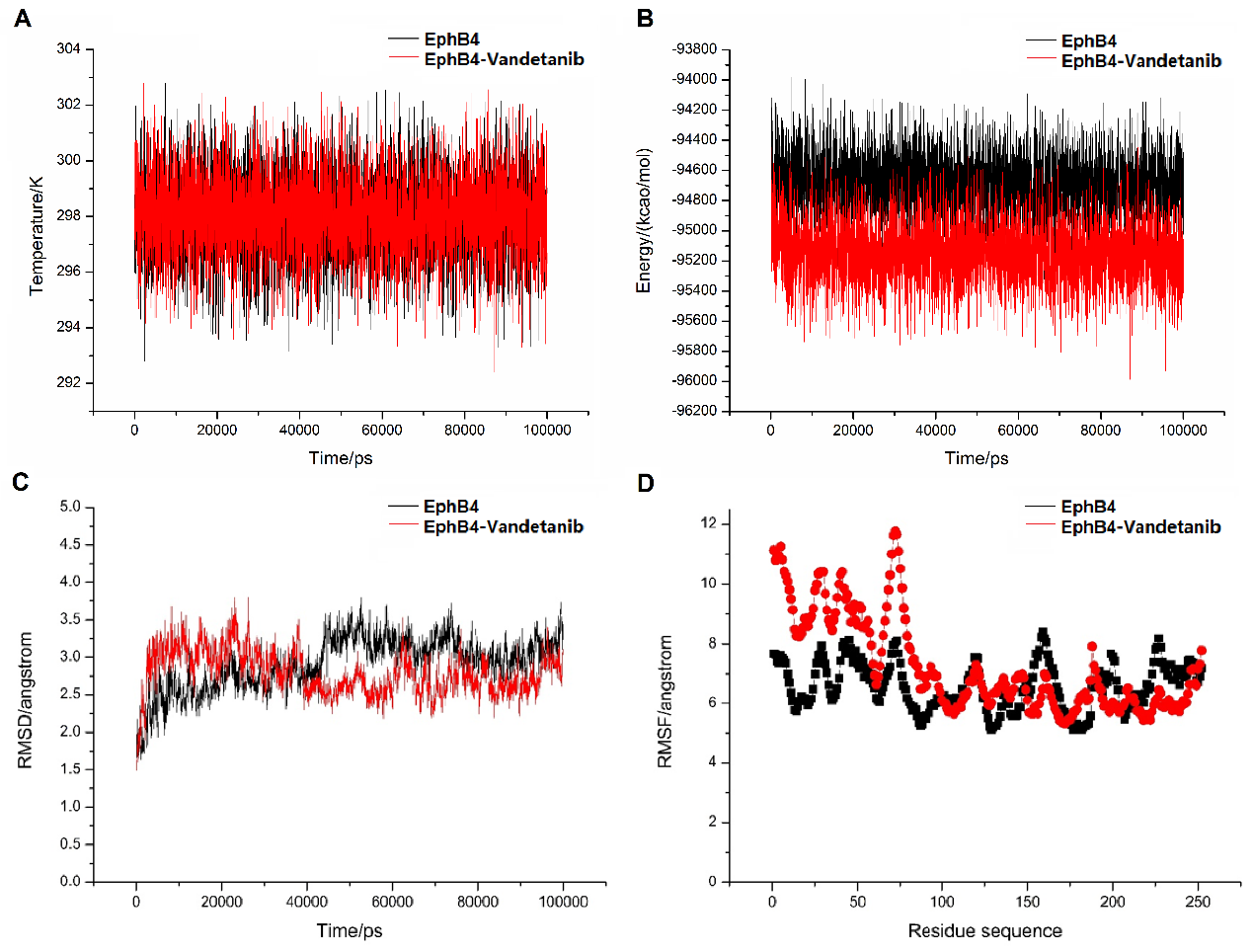


**Fig. S13** The stability of molecular dynamics simulation system. **(A)** The temperature variation of the models during 100 ns MD simulations. **(B)** The energy variation of the models during 100 ns MD simulations. **(C)** The backbone heavy-atom root-mean-square deviations (RMSD) of the EphB4 and EphB4-vandetanib complex at 298 K during 100 ns MD simulations. **(D)** The backbone atomic root-mean-square fluctuations (RMSF) values of the EphB4 and EphB4-vandetanib complex at 298 K during 100 ns MD simulations.


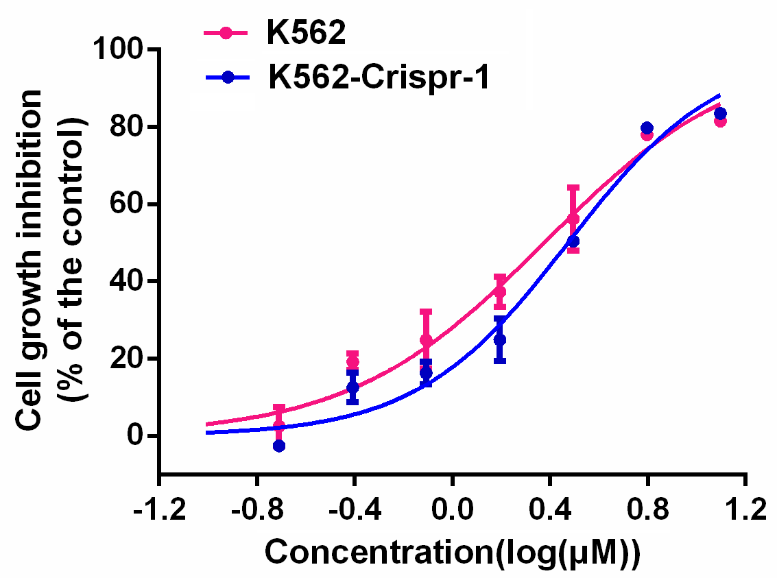


**Fig. S14** Vandetanib sensitivity analysis on wild type, EphB4-Crispr-1 cell lines after treatment for 48 h.


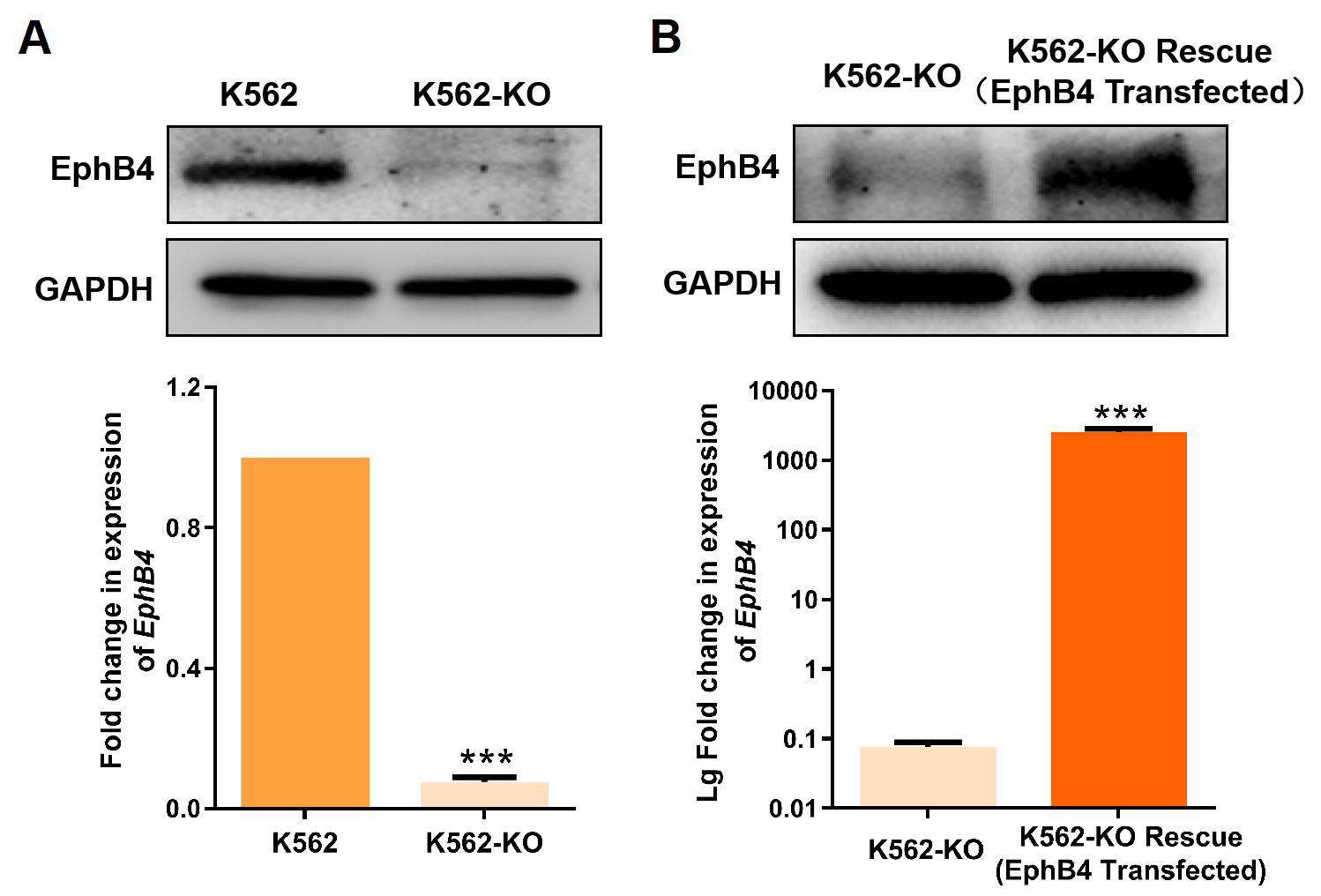


**Fig. S15** The mRNA and protein level of EphB4 on different cell lines. **(A)** The mRNA and protein level of EphB4 on K562 and K562-KO cell lines. ****p*< 0.001 versus the K562 group. **(B)** The mRNA and protein level of EphB4 on K562-KO and K562-KO Rescue cell lines. ****p*< 0.001 versus the K562 group.


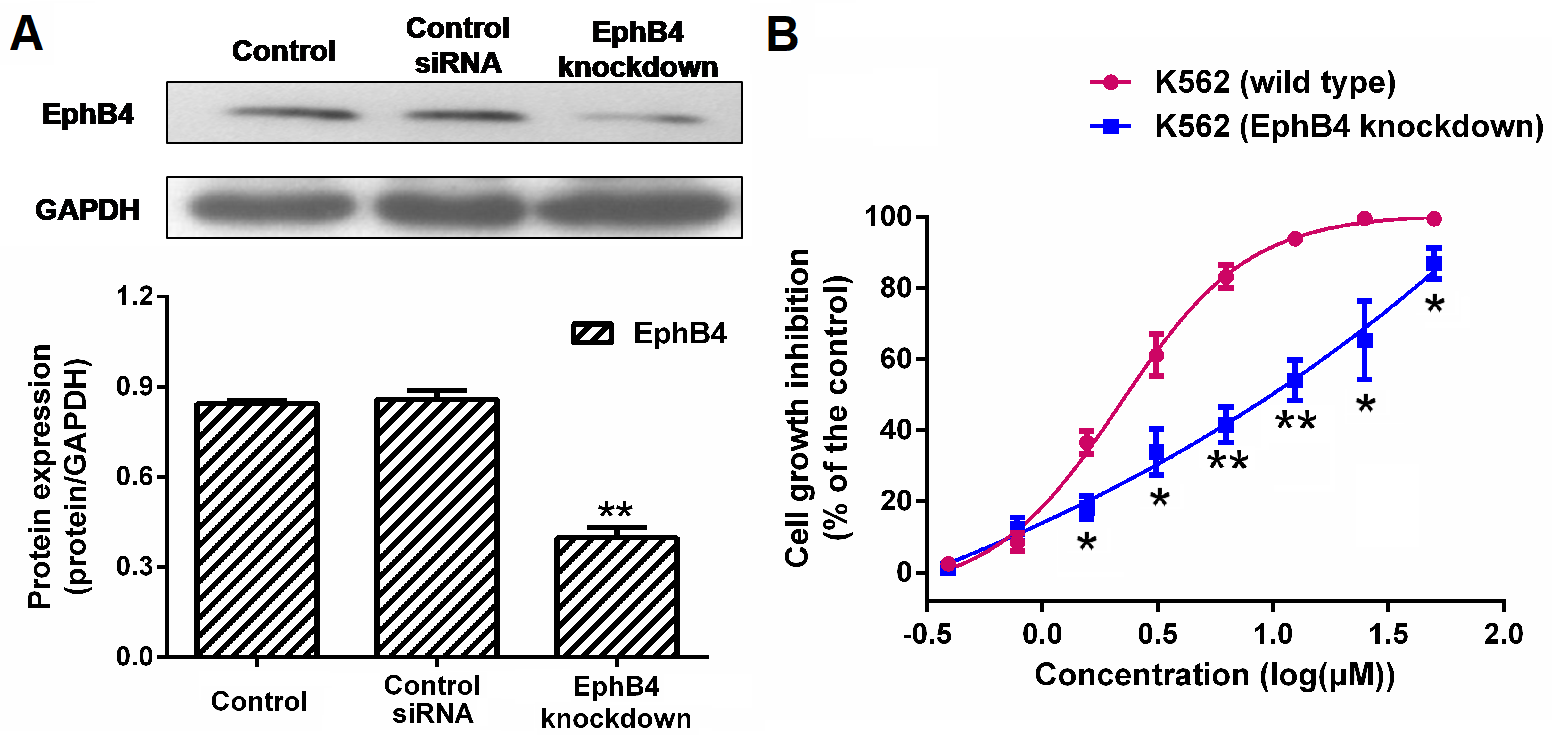


**Fig. S16** The role of EphB4 in the biological activity of vandetanib treatment. (A) EphB4 protein expression in wild‑type, Control siRNA, EphB4 knockdown K562 cells were determined by western blot analysis. Cells transfected with a control siRNA construct served as negative controls. Samples are derived from the same experiment, and blots were processed in parallel. (B) An MTT assay was used to evaluate the effect of vandetanib on cell proliferation in wild‑type and EphB4 knockdown K562 cells. * *p* < 0.05, ** *p* < 0.01 versus the control. Data represents the means ± SEM (n=3).


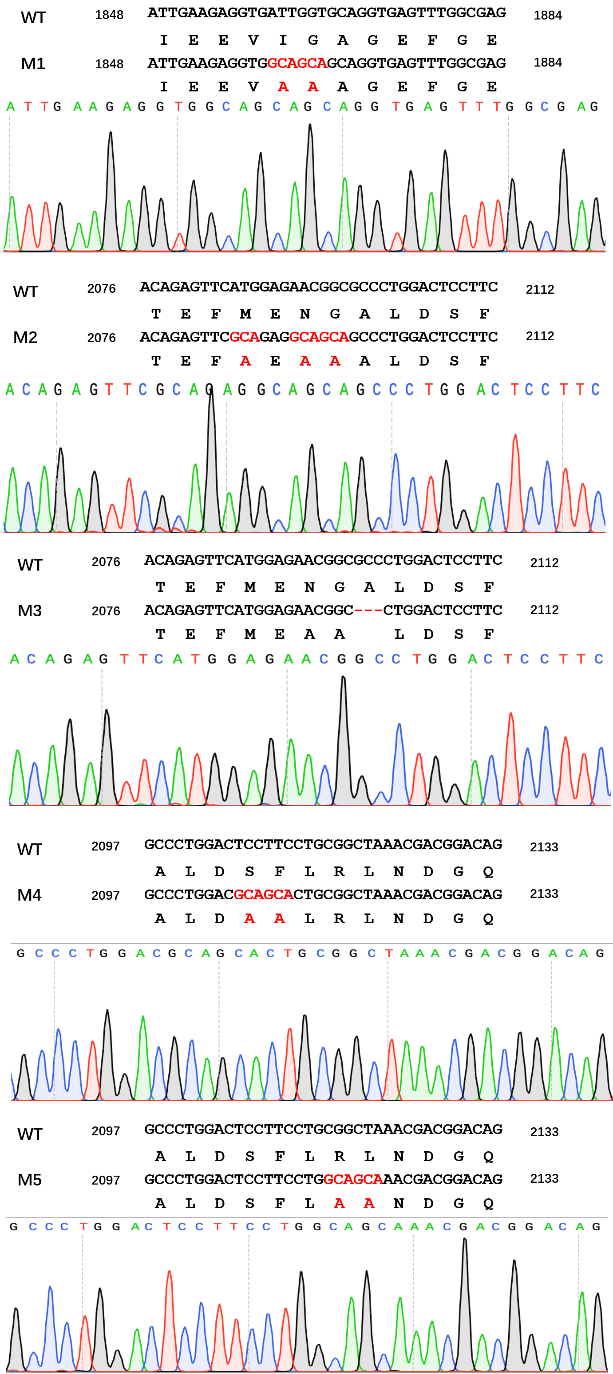


**Fig. S17** Sanger sequencing data of five point-mutation lines(M1-M5) and the corresponding WT sequences. The point-mutations of EphB4: M1, I14A, G15A; M2, M83A, N85A, G86A; M3, A87Del; M4, S90A, F91A; M5, R93A, L94A.


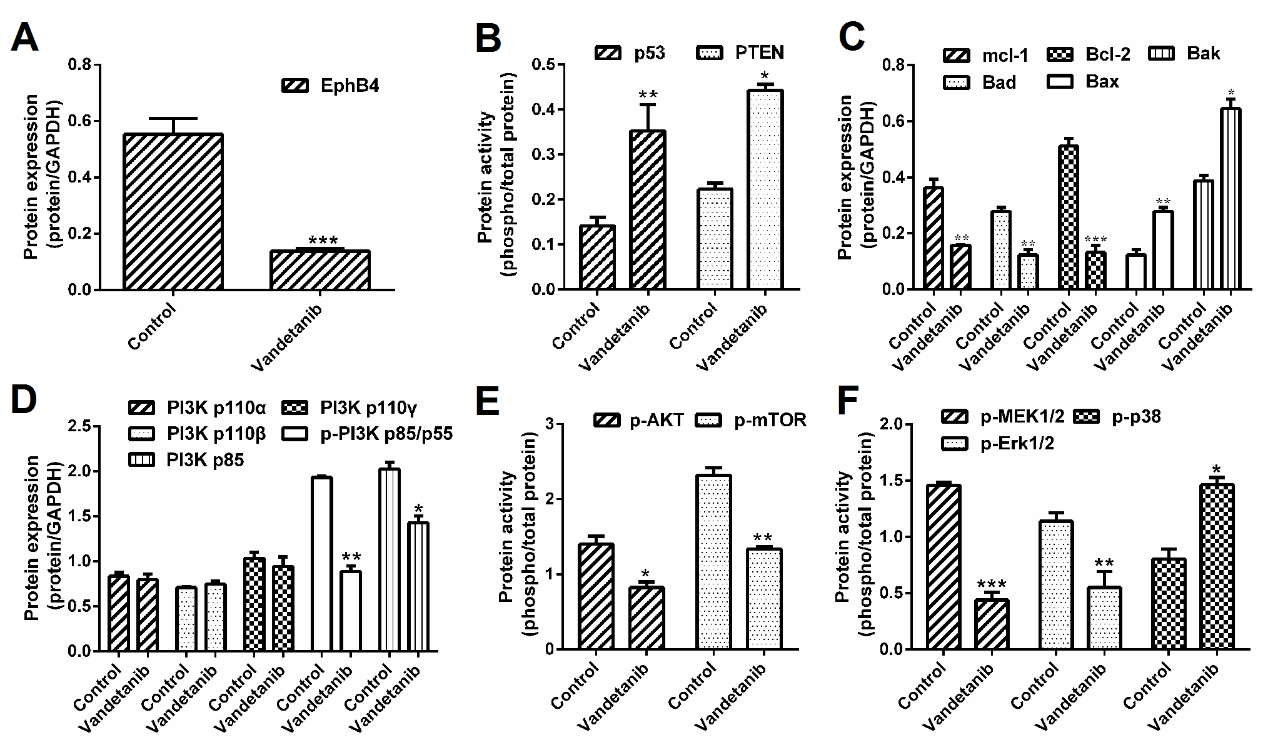


**Fig. S18** The results of western blot were quantified by densitometry analysis of the bands and normalization to GAPDH. **(A)** EphB4 protein in K562 tumor tissues. *** *p* < 0.001 versus the control. **(B)** The p53 and PTEN protein in K562 tumor tissues. * *p* < 0.05, ** *p* < 0.01 versus the control. **(C)** Apoptosis related protein in K562 tumor tissues. * *p* < 0.05, ** *p* < 0.01, *** *p* < 0.001 versus the control. **(D)** PI3K-p110α, PI3K-p110β, PI3K-p110γ, p-PI3K-p85/p55 and PI3K-p85 in K562 tumor tissues. * *p* < 0.05, ** *p* < 0.01 versus the control. **(E)** AKT and mTOR in K562 tumor tissues. * *p* < 0.05, ** *p* < 0.01 versus the control. **(F)** phosphorylation of MEK1/2, Erk1/2 and p38 in K562 tumor tissues. * *p* < 0.05, ** *p* < 0.01, *** *p* < 0.001 versus the control. Data represents the means ± SEM (n=3).

**2. Supplementary Tables**

**Table S1** The information about the antibody used in western blot assay

| Antibody name | Polyclonal/  monoclonal | Source | Catalog No./  clone No. |
| --- | --- | --- | --- |
| mcl-1 | monoclonal | Protein Technology Group | 16225-1-AP |
| Bad | monoclonal | Protein Technology Group | 10435-1-AP |
| Bcl-2 | monoclonal | Protein Technology Group | 12789-1-AP |
| Bax | monoclonal | Protein Technology Group | 50599-2-lg |
| Bak | monoclonal | Cell Signaling | 12105 |
| EphB4 | monoclonal | Cell Signaling | 14960 |
| p-EphB4 | monoclonal | Affinity Biosciences LTD | AF8432 |
| p38 | monoclonal | Cell Signaling | 8690 |
| p-p38 | monoclonal | Cell Signaling | 4511 |
| p53 | monoclonal | Cell Signaling | 2527 |
| PTEN | monoclonal | Cell Signaling | 9188 |
| Rac1 | monoclonal | Cell Signaling | 4651 |
| Erk1/2 | monoclonal | Cell Signaling | 4370 |
| p-Erk1/2 | monoclonal | Cell Signaling | 3510 |
| MEK1/2 | monoclonal | Cell Signaling | 3958 |
| p-MEK1/2 | monoclonal | Cell Signaling | 9154 |
| AKT | monoclonal | Cell Signaling | 4691 |
| p-AKT | monoclonal | Cell Signaling | 9611 |
| mTOR | monoclonal | Cell Signaling | 2983 |
| p- mTOR | monoclonal | Cell Signaling | 5536 |
| PI3K p85 | monoclonal | Cell Signaling | 4292 |
| p-PI3K p85 | monoclonal | Cell Signaling | 4228 |
| PI3K p110α | monoclonal | Cell Signaling | 4255 |
| PI3K p110β | monoclonal | Cell Signaling | 3011 |
| PI3K p110γ | monoclonal | Cell Signaling | 4252 |
| GAPDH | monoclonal | Protein Technology Group | 10494-1-AP |
| goat anti-rabbit IgG | monoclonal | Protein Technology Group | 10285-1-AP |

**Table S2** Primers sequences

| Primers | Sequence |
| --- | --- |
| Human GAPDH | F: 5'-GCACCGTCAAGGCTGAGAAC-3'  R: 5'-TGGTGAAGACGCCAGTGGA-3' |
| Human EphB4 | F: 5'-CAGGAACATCACAGCCAGAC-3'  R: 5'-CAGGACCAGGACCACACC-3' |
| Mouse GAPDH | F: 5'-AGGTTGTCTCCTGCGACTTCA-3' |
|  | R: 5'-TGGTCCAGGGTTTCTTACTCC-3' |
| Mouse EphB4 | F: 5'-TATGCCACGATACGCTTCAC-3' |
|  | R: 5'-CGCTTCCGAGTCAGATGTTC-3' |

**Table S3** Primers and oligos sequences

| Primers/oligos | Sequence |
| --- | --- |
| EphB4-Crispr-1 | F：caccGAAGAGGTGCAGGGATAGCA  R：aaacTGCTATCCCTGCACCTCTTC |
| EphB4-Crispr-2 | F：caccGGGCGCACTTTTTGTAGAAG  R：aaacCTTCTACAAAAAGTGCGCCC |

**Table S4** The K562 cell growth inhibition by compounds (10 μM) screened in this study

| Compounds | Inhibition rate (%, 10 μM) | | | Compounds | Inhibition rate (%, 10 μM) | | Compounds | | Inhibition rate (%, 10 μM) | |
| --- | --- | --- | --- | --- | --- | --- | --- | --- | --- | --- |
| vandetanib | | 99.71 | arteannuic acid | | | -69.83 | | pazopanib | | 76.24 |
| afatinib | | 78.59 | artemisinin | | | -161.25 | | cabozantinib | | 90.10 |
| lapatinib | | 98.79 | imperatorin | | | -7.73 | | fenoterol | | -62.04 |
| sorafenib | | 73.38 | isoimperatorin | | | -85.88 | | terbutaline | | -58.17 |
| sunitinib | | 73.47 | dictamnine | | | -0.51 | | salbutamol | | -65.78 |
| erlotinib | | 3.52 | salvianolic acid B | | | -40.83 | | tuloterol | | -24.54 |
| regorafenib | | 8.49 | cyproheptadine | | | -38.42 | | clorprenaline | | -36.16 |
| nilotinib | | 71.70 | xanthotoxin | | | -42.22 | | bambuterol | | -43.13 |
| gefitinib | | -93.35 | sinomenine | | | -4.70 | | salmeterol | | -72.70 |
| practolol | | -62.13 | psoralen | | | -51.46 | | maraviroc | | -56.26 |
| sotalol | | -52.78 | metipranolol | | | -26.75 | | bupropion | | -48.47 |
| tamsulosin | | -35.24 | brevibloc | | | -12.58 | | domperidone | | -38.58 |
| doxazosin | | -84.45 | nifedipine | | | 6.88 | | amantadine | | -58.77 |
| terazosin | | -57.01 | nimodipine | | | -69.66 | | valaciclovir | | -63.89 |
| alfuzosin | | -37.25 | nitrendipine | | | -26.35 | | zidovudine | | 10.33 |
| phentolamine | | -43.12 | nicardipine | | | -74.23 | | sustiva | | -40.09 |
| berberine | | 49.80 | amlodipine | | | -59.79 | | saquinavir | | -30.40 |
| dihydroberberine | | 30.63 | baicalin | | | -30.98 | | acyclovir | | -42.56 |
| homoharringtonine | | 68.69 | diphenhydramine | | | -37.90 | | indinavir | | -52.19 |
| rhynchophylline | | -47.32 | chlorpheniramine | | | -42.86 | | nevirapine | | -46.95 |
| proguanil | | -7.92 | mizolastine | | | -89.13 | | oseltamivir | | -47.65 |
| salvianolic acid A | | -32.30 | doxepin | | | -54.91 | | valsartan | | -32.43 |
| brucine | | -63.48 | salvianolic acid C | | | -2.73 | | telmisartan | | -8.14 |
| cryptotanshinone | | -72.92 | azelastine | | | -74.26 | | losartan | | -72.89 |
| morphine | | -46.72 | dopamine | | | -62.26 | | candesartan | | -49.70 |
| heroin | | -30.23 | olanzapine | | | -53.49 | | irbesartan | | -56.40 |
| ciprofloxacin | | -3.17 | quetiapine | | | -46.23 | | olmesartan | | -34.82 |
| codeine | | -63.96 | verapamil | | | 66.00 | | lidocaine | | -38.48 |
| thebaine | | -58.34 | hydroxysafflor | | | -61.07 | | ephedrine hydrochloride | | -29.00 |
| pethidine | | -43.68 | quercetin | | | -58.40 | | isoliquiritigenin | | -122.99 |
| methadone | | -33.43 | sophocarpidine | | | -62.65 | | neohesperidin | | -49.43 |
| arteannuin b | | -8.46 | tetrahydropalmatine | | | -83.41 | | emodin | | 6.90 |
| vanillin | | -86.16 | cyasterone | | | -50.56 | | hedera helix | | -52.41 |
| arctiin | | -62.65 | bupleurin A | | | 28.49 | | osthole | | -21.25 |

**Table S5** The IC_50_ of TKIs in K562 cells and H9 cells

| Compounds | IC_50_ (μM) | |
| --- | --- | --- |
|  | K562 cells | H9 cells |
| Vandetanib | 2.18±1.07 | 11.24±1.08** |
| Afatinib | 8.62±1.09^##^ | 22.59±1.03** |
| Lapatinib | 7.69±1.03^##^ | 16.00±1.04** |

Note: **p*< 0.05, ***p*< 0.01 versus the K562 group. ^#^*p*< 0.05, ^##^*p*< 0.01 versus the vandetanib group.

**Table S6** The IC_50_ of vandetanib in K562 cells, MEG-01 cells and H9 cells

| Time | IC_50_ (μM) | |  |
| --- | --- | --- | --- |
|  | K562 cells | H9 cells | MEG-01 cells |
| 24 h | 43.31±2.14 | 17.19±1.05** | 1.94±1.26** |
| 48 h | 2.18±1.07 | 11.24±1.08** | 1.46±1.07 |
| 72 h | 0.90±0.50 | 8.57±3.67* | 0.82±0.68 |

Note: **p*< 0.05, ***p*< 0.01 versus the K562 group.

**Table S7** The *K*_D_ values of TKIs interacting with EphB4 by SPR analysis

| Compounds | *K*_D_ value (μM) | |
| --- | --- | --- |
|  | ag10042 | ag16996 |
| Vandetanib | 1.48 | 4.65 |
| Afatinib | 3.87 | 3.75 |
| Lapatinib | 5.40 | 12.14 |
| NVP-BHG712 | 3.85 | 4.75 |

**Table S8** The IC_50_ of TKIs on EphB4 kinase activity by kinase assay

| Compounds | IC_50_ (nM) |
| --- | --- |
| Vandetanib | 68.33±1.03 |
| Afatinib | 1708±1.04*** |
| Lapatinib | >100000*** |
| NVP-BHG712 | 349.30±1.06*** |

Note: ****p*< 0.001 versus the vandetanib group.

**Table S9** The predicted binding free energies and the individual energy components computed by MM/PBSA method (neglecting the configurational entropy, kcal/mol)

| Complex | ∆*E*_ele_ | ∆*E*_vdw_ | ∆*G*_np_ | ∆*G*_ele_ | ∆*G*_solv_ | ∆*G*_bind_ |
| --- | --- | --- | --- | --- | --- | --- |
| EphB4-vandetanib | -9.35  ±3.43 | -32.95  ±4.11 | -4.95  ±0.49 | 11.11  ±2.28 | 15.50  ±3.15 | -26.80  ±3.70 |

**Table S10** The IC_50_ of vandetanib in K562 cell line and other mutant sublines

| Cell lines | IC_50_ (μM) |
| --- | --- |
| K562-WT | 2.05±1.08 |
| K562-KO | 3.94±1.08 |
| K562-Rescue | 1.61±1.09 |
| K562-M1 | 1.93±1.08 |
| K562-M2 | 1.71±1.08 |
| K562-M3 | 1.10±1.09 |
| K562-M4 | 2.59±1.07 |
| K562-M5 | 2.94±1.08 |
